# How to reduce sitting time? A review of behaviour change strategies used in sedentary behaviour reduction interventions among adults

**DOI:** 10.1080/17437199.2015.1082146

**Published:** 2015-09-16

**Authors:** Benjamin Gardner, Lee Smith, Fabiana Lorencatto, Mark Hamer, Stuart JH Biddle

**Affiliations:** ^a^ Department of Psychology, Institute of Psychiatry, Psychology and Neuroscience (IoPPN), King's College London, London, UK; ^b^ UCL Centre for Behaviour Change, University College London, London, UK; ^c^ Health Behaviour Research Centre, University College London, London, UK; ^d^ Health Services Research & Management Division, School of Health Sciences, City University London, London, UK; ^e^ National Centre for Sport and Exercise Medicine, Loughborough University, Loughborough, UK; ^f^ Institute of Sport, Exercise & Active Living, Victoria University, Melbourne, Australia; ^g^ The NIHR Leicester-Loughborough Diet, Lifestyle and Physical Activity Biomedical Research Unit, Loughborough, UK

**Keywords:** sedentary behaviour, behaviour change, intervention

## Abstract

Sedentary behaviour – i.e., low energy-expending waking behaviour while seated or lying down – is a health risk factor, even when controlling for physical activity. This review sought to describe the behaviour change strategies used within interventions that have sought to reduce sedentary behaviour in adults. Studies were identified through existing literature reviews, a systematic database search, and hand-searches of eligible papers. Interventions were categorised as ‘very promising’, ‘quite promising’, or ‘non-promising’ according to observed behaviour changes. Intervention functions and behaviour change techniques were compared across promising and non-promising interventions. Twenty-six eligible studies reported thirty-eight interventions, of which twenty (53%) were worksite-based. Fifteen interventions (39%) were very promising, eight quite promising (21%), and fifteen non-promising (39%). Very or quite promising interventions tended to have targeted sedentary behaviour instead of physical activity. Interventions based on environmental restructuring, persuasion, or education were most promising. Self-monitoring, problem solving, and restructuring the social or physical environment were particularly promising behaviour change techniques. Future sedentary reduction interventions might most fruitfully incorporate environmental modification and self-regulatory skills training. The evidence base is, however, weakened by low-quality evaluation methods; more RCTs, employing no-treatment control groups, and collecting objective data are needed.

Sedentary behaviour has been defined as any waking behaviour characterised by an energy expenditure of 1.5 metabolic equivalents or less, undertaken while sitting or lying down (Sedentary Behaviour Research Network [SBRN], 2012). It is increasingly recognised as a risk factor for mortality and morbidity, after controlling for moderate-to-vigorous physical activity (Wilmot et al., 2012). Self-report data suggest the average European adult spends 5 hours sitting per weekday (Bennie et al., 2013). Among UK office workers, the figure may be higher: using objective data, one study showed 5.3 hours of the working day to be spent sitting (Ryan, Grant, Dall, & Granat, 2011), and another that workers spend 10.6 weekday hours sitting on average between 7 am and 11 pm (Smith et al., 2015). While those who spend more time in sedentary behaviour tend to do less moderate-to-vigorous physical activity (Mansoubi, Pearson, Biddle, & Clemes, 2014), the potentially independent health impacts of sedentary behaviour and physical activity mean that activity-promotion may fail to offset the health impact of sedentary behaviour (Henson et al., 2013).

Sedentary behaviour reduction interventions have been conducted among children for many years (Biddle, Gorely, & Stensel, 2004), but interventions among adults are much more recent, and more are needed. Syntheses of previous intervention trials can provide a valuable input into the design of new interventions, by revealing which approaches, techniques, and assumptions show promise in reducing sedentary behaviour, and the strength of the evidence (Craig et al., 2008; Michie, Atkins, & West, 2014). To our knowledge, only four syntheses of sedentary behaviour reduction interventions have been undertaken to date (Chau et al., 2010; Owen et al., 2011; Prince, Saunders, Gresty, & Reid, 2014; Shrestha, Ijaz, Kukkonen-Harjula, Kumar, & Nwankwo, 2015), of which two focused on worksite-based interventions only (Chau et al., 2010; Shrestha et al., 2015). Two of the four reviews reported meta-analyses of intervention effects (Prince et al., 2014; Shrestha et al., 2015), and concluded that, while the quality of included studies was at best moderate, interventions that target sedentary behaviour have the potential to decrease it. Yet, effect sizes varied, with intervention recipients in one study achieving a reduction of 176 min/day of sedentary behaviour, and in another a decrease of only 52 min/day. No review to date has focused on the discrete behaviour change techniques that may distinguish more effective from less effective interventions.

Developing effective sedentary reduction interventions depends on understanding both what works in changing sedentary behaviour and why. Recent advances in behavioural science permit the identification of intervention components that may explain between-study variation in effectiveness (e.g., Michie & Prestwich, 2010; Michie et al., 2013). A recent framework (the ‘Behaviour Change Wheel’; Michie, van Stralen, & West, 2011) proposes that interventions can play one or more of nine functions in order to change behaviour; for example, interventions may seek to *educate* the target population of the need for change, *persuade* them by inducing positive or negative emotions around the behaviour, or *train* them in the skills needed to achieve change. A taxonomy is available which describes 93 discrete behaviour change techniques that may be used in interventions within any behavioural domain (e.g., providing information on health consequences, setting goals, restructuring the physical environment; Michie et al., 2011). Intervention functions represent ‘broad categories of means by which an intervention can change behaviour’ (Michie, Atkins, & West, 2014, p. 109), and behaviour change techniques represent the observable and irreducible intervention components that serve to perform one or more functions (Michie & West, 2013). Coding for intervention functions and behaviour change techniques in published intervention descriptions can provide a useful summary of the broad strategies and specific techniques that have previously been employed, and comparing these components across effective and ineffective interventions can point towards the possible ‘active ingredients’ of interventions (Gardner, Whittington, McAteer, Eccles, & Michie, 2010; Gilinsky et al., 2015; Martin, Chater, & Lorencatto, 2013; Michie, Abraham, Whittington, McAteer, & Gupta, 2009). Interventions based on theory can be more effective (Ivers et al., 2012; but see Gourlan et al., 2014), and where theory use is scant, identifying the intervention functions and behaviour change techniques used can reveal the implicit theoretical assumptions underpinning interventions (Gardner et al., 2010). For example, providing information on the health impact of sitting assumes sedentary behaviour is driven by a lack of knowledge, and that increasing knowledge will change behaviour (Abraham & Michie, 2008). Where techniques are found to be associated with promising interventions, this can inform hypothesising around the possible psychological or other pathways through which sedentary behaviour might best be reduced. Conversely, identifying strategies associated with less promising interventions can ensure that intervention designers do not devote time and resources to developing unhelpful strategies.

The aim of this review was to consider how sedentary behaviour in adults might best be reduced, by describing the behaviour change strategies used in sedentary behaviour reduction intervention evaluations. Our review goes beyond previous evidence syntheses in this field by exploring intervention components that may act as potential sources of variation in effects. Given the relative infancy of the sedentary behaviour change field, our review does not aim to provide definitive conclusions regarding the most effective intervention components; rather, it is designed to offer input into the development of future sedentary reduction interventions, by highlighting which behaviour change strategies have shown promise in previous studies (e.g., Craig et al., 2008; Michie, Atkins, & West, 2014). We treated interventions as ‘promising’ where sedentary behaviour was observed to have reduced on at least one measure. We coded for intervention characteristics, to identify which functions and techniques have been used to reduce sedentary behaviour and which were more associated with potential for achieving reduction in sedentary behaviour, and study-level methodological characteristics for descriptive purposes only. We included any intervention for which evidence was available regarding extent of change in sedentary behaviour, regardless of whether sedentary behaviour change was an explicit target. The protocol for this review is not publicly available, nor was the review registered on the PROSPERO database. Nonetheless, relevant PRISMA systematic review guidelines were followed (Moher, Liberati, Tetzlaff, & Altman, 2009). A completed PRISMA checklist is available as supplemental material.

## Methods

### Selecting papers for review

#### Eligibility criteria

Studies were included where they met the following criteria (Schardt, Adams, Owens, Keitz, & Fontelo, 2007). The study *population* was adults aged 18 or over, recruited from the general population. Clinical populations – i.e., participants recruited on the basis of their membership of a clinical group, such as having diabetes – were excluded. Overweight and obese participants were not considered a clinical population.

The eligibility of *interventions* was dependent on *outcomes*, such that any behaviour change intervention was eligible where primary quantitative data were available pertaining to pre-post changes in at least one indicator of sedentary behaviour among those receiving the intervention. Interventions that did not explicitly target sedentary behaviour were thus included where pre- and post-intervention sedentary behaviour data were available. Objective and self-report data were entered into the review. Objective data were taken as indicative of sedentary behaviour only where based on direct observation, or a combined accelerometer–inclinometer device sensitive to both activity and posture, so as to reliably differentiate between sitting or lying down and standing (Grant, Ryan, Tigbe, & Granat, 2006). Sedentary behaviour objectively measured in this way was defined as time spent in minimal energy expenditure, in a seated or reclined position (SBRN, 2012). Studies in which sedentary behaviour was objectively estimated only as low physical activity (e.g., low step count) were excluded (e.g., Andersen, Høstmark, Holme, & Anderssen, 2013; Gardiner, Eakin, Healy, & Owen, 2011; Gilson, Suppini, Ryde, Brown, & Brown, 2012), as this depicts inactivity, not sedentary behaviour, identification of which depends on postural allocation methods. The only eligible self-report sedentary behaviour indices were sitting time (total waking sitting or domain-specific, e.g., worksite sitting), time spent lying down while awake, or a combination thereof, though the latter two indices were not found in any study. While sedentary behaviour has previously been operationalised as time spent in activities typically done while seated (e.g., TV viewing, computer use; Gardiner, Clark, et al., 2011; Rhodes, Mark, & Temmel, 2012), studies in which only these behaviours were measured were excluded, because they may be performed while active and so do not reliably denote true sedentary behaviour (e.g., Rovniak et al., 2014). The eligible *type of study* was peer-reviewed, and published in full text in English. No eligibility criteria were based on *comparisons* or *type of question asked*.

#### Search procedure

Three search strategies were used. First, potentially eligible references were identified from two existing reviews of sedentary behaviour reduction interventions (Chau et al., 2010; Owen et al., 2011). (Two more recent reviews were published after our search was completed [Prince et al., 2014; Shrestha et al., 2015].) Second, an electronic search of seven databases (CINAHL Plus, Embase, ISI Web of Knowledge, MEDLINE, PsycArticles Full Text, PsycInfo, SPORTDiscus with Full Text) was undertaken on 25 April 2014. Search filters were applied to each database to specify: interventions to reduce sedentary behaviour or increase physical activity; intervention evaluation designs; and exclusion of non-adult and clinical samples. No date limits were set. Example search terms are provided in Table A1 in Supplemental material. Third, reference lists of eligible records thus identified were hand-searched for additional papers.

#### Search results and screening

Searches and screening were undertaken by a health psychologist fully trained in database searching and experienced in systematic reviewing (BG). To estimate screening reliability, a physical activity epidemiologist (LS) independently screened the titles and abstracts of 20% of all records returned by the search procedure, and full texts of 20% of records selected for full-text assessment by the first coder. Title and abstract agreement, judged according to whether the selected papers incorporated those selected by the first coder for inclusion, was 100%, and full-text agreement 98%. Disagreement on one full-text record (2%) was resolved through discussion.

**Figure 1.**
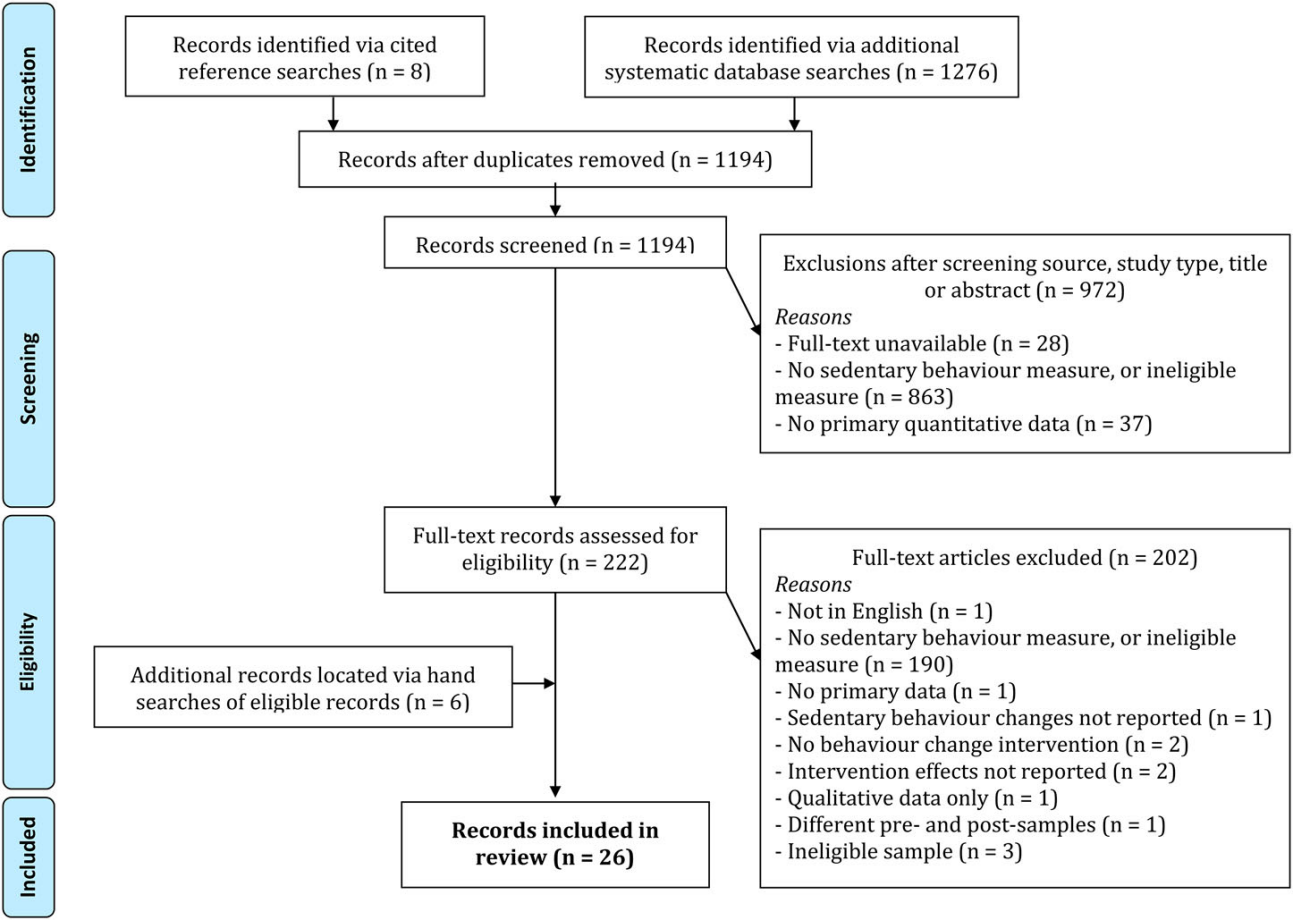
Search strategy and screening procedure.

Eight papers were identified from previous reviews (see Figure 1). Database searches returned 1194 unique records, of which 20 were eligible, from the reference lists of which a further 6 eligible papers were identified. The final dataset comprised 26 papers.

#### Additional materials

Corresponding authors were emailed and asked to provide additional information for coding. Twenty-three authors were approached (including three corresponding for multiple papers), for two (9%) of whom email addresses were no longer functioning and could not be traced online. Thirteen (57%) did not respond. Of eight authors (35%) who did respond, three (13% of all authors) stated that no more information was available, and five (22%) sent additional material.

### Data extraction

Alongside information from the 26 eligible papers, additional detail of the intervention treatment(s) was coded from: records in open-access trial registries for seven papers that reported a trial registration reference code; published supplementary materials (two papers); one or more linked publications cited in the included paper (five papers); and published and unpublished material provided by authors (four and two papers, respectively).

At least 32 data segments were extracted from all material relevant to each paper, with an additional 14 segments extracted for each additional intervention or control group. A data extraction form was developed, applied and iteratively refined by a health psychologist (BG) to ensure adequate data capture. A second health psychologist (FL) independently coded all available material for seven (27%) randomly selected papers, using the final data extraction form (Version 9). Inter-coder agreement was assessed using percentage agreement and kappa (*ĸ*). Kappa values were interpreted according to Landis and Koch's (1977) criteria, whereby *ĸ* > 0 < .20 denoted slight, *ĸ* ≥ .20 < .40 fair, *ĸ* ≥ .40 < .60 moderate, *ĸ* ≥ .60 < .80 substantial, and *ĸ* ≥ .80 almost perfect agreement. Discrepancies were resolved through discussion.

#### Study characteristics

*Methodological characteristics* extracted included country, setting, study design, length of follow-up(s), the number of arms and interventions, theory basis, and whether the extent of prior sedentary behaviour was an eligibility criterion. We coded treatments as ‘interventions’ where they involved any attempt to modify any behaviour (physical activity, sedentary behaviour, other), to allow for the possibility that sedentary behaviour may change even where not explicitly targeted. Groups described as ‘control groups’ in published papers were therefore coded as intervention groups where they received a behaviour change treatment. Advice to maintain current behaviour was not deemed a behaviour change treatment. An extensive coding frame was used to code the theory basis of interventions (Michie & Prestwich, 2010), but data could only be consistently extracted for only one item, relating to whether a named theory of behaviour or behaviour change was mentioned in the Abstract, Introduction or Method section. Prior sedentary behaviour was coded as an eligibility criterion where explicitly stated as such, and where participants were selected on the basis of observed sedentary behaviour levels, or employment in a sedentary occupation (e.g., seated deskwork). Inter-rater reliability for all methodological characteristics was perfect (100%; *ĸ* = 1).

*Sample characteristics* extracted were participant description (e.g., employees, parents), and, for each group, baseline and follow-up sample size, and demographics (age, gender). Inter-rater reliability was perfect (100% agreement; *ĸ* = 1).

*Study quality* was coded using an adaptation of the quality assessment tool used in Chau et al's (2010) review of sedentary reduction interventions, which itself was adapted from a checklist developed, through expert consensus, to capture minimum quality standards for intervention trials (Verhagen et al., 1998). This tool was chosen to allow our readers to compare quality scores for studies in this review with those reported by Chau et al. (2010). Items covered were the following: randomisation method, treatment allocation concealment, similarity of groups at baseline on physical activity or sedentary behaviour, specification of eligibility criteria, assessor blinding, evidence of point estimates and validity of at least one of the sedentary behaviour measures used to assess intervention promise, and presence of an intention to treat analysis.^1^ Each of the seven items was coded as yes, no, unclear, or not applicable, and a score of 1 was allocated for each ‘yes’ response, and 0 for all other responses, producing a 0–7 quality index. This scoring system restricted single-arm study designs to a maximum score of 3, as only three items were applicable (eligibility criteria, point estimate and validity, and intention to treat). Inter-rater reliability was substantial (90% agreement; *ĸ* = .80).

*Outcome data* extracted related to whether sedentary behaviour was self-reported or objectively measured, and, for each group, whether statistically significant within- or between-group changes were found on any measure of sedentary behaviour at any follow-up point. Inter-rater reliability was almost perfect (97%; *ĸ* = .93).

#### Intervention characteristics

Intervention characteristics, extracted for each treatment, related to the behaviours explicitly targeted (e.g., physical activity, sedentary behaviour), the behaviour change that was the primary aim of the intervention (e.g., to increase physical activity), and the intervention functions and behaviour change techniques used. The primary behaviour change aim was coded from explicit statements of intervention purpose where possible. Where no such statement was available, interventions were assumed to have primarily targeted physical activity, not sedentary behaviour. To ensure data were extracted where sedentary behaviour was not an explicit target, functions and techniques were coded where used to target sedentary behaviour and/or physical activity; this ensured that data were extracted where sedentary behaviour change was not an explicit intervention target.

*Intervention functions.* Each intervention was coded as performing one or more of nine functions, using descriptions taken from the Behaviour Change Wheel (Michie et al., 2011, p. 7): education (‘increasing knowledge or understanding’), persuasion (‘using communication to induce positive or negative feelings or stimulate action’), incentivisation (‘creating expectation of reward’), coercion (‘creating expectation of punishment or cost’), training (‘imparting skills’), restriction (‘using rules to reduce the opportunity to engage in the target behaviour [or to increase the target behaviour by reducing the opportunity to engage in competing behaviours]’), environmental restructuring (‘changing the physical or social context’), modelling (‘providing an example for people to aspire to or imitate’), and enablement (‘increasing means/reducing barriers to increase capability or opportunity beyond environmental restructuring’). Inter-rater reliability for intervention functions was substantial (83% agreement, *ĸ* = .67).

*Behaviour change techniques.* The Behaviour Change Technique Taxonomy v1, a reliable 93-item coding frame (Michie et al., 2013), was used to identify and characterise techniques present in intervention and comparator treatment descriptions. Each of the 93 behaviour change techniques was given a global rating as either present (1) or absent (0). The frequency with which techniques were delivered was not coded. Both coders have extensive experience of coding behaviour change techniques, having coded techniques for published reviews (e.g., Gardner et al., 2010; Martin et al., 2013), and trained other coders in tutorials, organised by Michie and colleagues, on applying the Behaviour Change Technique Taxonomy v1 to intervention descriptions (see Wood et al., 2015). To avoid inflating reliability due to agreed absence of most behaviour change techniques, a conservative estimate was made based only on techniques identified as present by either coder. Reliability was substantial (92% agreement, *ĸ* = .83).

### Analysis strategy

Given that outcome data indicating change in sedentary behaviour were purposefully selected to assess intervention potential, meta-analysis was not appropriate. Instead, we divided interventions into three categories according to their apparent potential to reduce sedentary behaviour. Potential was judged according to whether within- or between-group analyses showed statistically significant reductions in sedentary behaviour at one or more follow-up points relative to baseline. Interventions were deemed ‘very promising’ where there were significant reductions in at least one sedentary behaviour indicator within the intervention group, *and* reduction on this indicator was greater than observed in at least one comparator arm (i.e., control, or another intervention). Interventions were deemed ‘quite promising’ where there were *either* significant declines in at least one sedentary behaviour indicator within the intervention group, *or* reduction in at least one sedentary behaviour indicator was greater than observed in at least one comparator arm. Interventions were deemed ‘non-promising’ where there were *neither* sedentary behaviour changes within the intervention arm *nor* differences in sedentary behaviour change relative to at least one comparator arm. This classification system was designed to ensure that interventions showing any promise were coded as such, and that those showing the strongest evidence of promise were distinguished from those with lesser evidence.

Descriptive and statistical analyses were undertaken to compare intervention characteristics according to our ratings of promise. Chi-square tests were run to assess whether interventions that explicitly sought to reduce sedentary behaviour (or to both reduce sedentary behaviour and increase physical activity) were more promising than those that did not. Associations between intervention promise and the number of intervention functions and techniques observed were examined using one-way ANOVAs, with two sets of planned comparisons, respectively, comparing very and quite promising against non-promising interventions, and quite promising against non-promising interventions. *T*-values and degrees of freedom were adjusted where Levene's test indicated heterogeneity of variance. One-tailed *p*-values are reported for all ANOVAs and t-tests.

The potential contribution of intervention functions and behaviour change techniques to intervention promise was judged using a ‘promise ratio’, which was calculated as the number of (very or quite) promising interventions featuring the function (or technique) divided by the number of non-promising interventions featuring the function (or technique) (Martin et al., 2013). Functions and techniques were deemed promising where used in at least twice as many promising as non-promising interventions (i.e., promise ratio ≥ 2), and in at least two interventions in total (to avoid over-interpreting scant data). Where functions or techniques were used only in (two or more) promising interventions (promise ratio = ∞), the number of interventions in which they were used was reported instead of the ratio.

Given considerable interest in the potential for reducing sedentary behaviour in the workplace (Chau et al., 2010; Shrestha et al., 2015), supplementary analyses were run for interventions conducted in worksite settings. No paper described both worksite and non-worksite interventions.

## Results

### Study characteristics

Table 1 summarises study characteristics, and Table A2 in Supplemental material reports further study detail. The 26 papers reported 26 studies, and 38 interventions. Twelve studies (46%) were conducted solely in Europe (including four undertaken in the UK), eight (31%) in North America (seven in the USA), and five (19%) in Australia. One study combined samples from the UK, Australia, and Spain. Fourteen studies (54%) were set in the workplace. Of the twelve non-worksite studies (46%), seven (27% of all studies) were set in the community, two (8%) were hosted online, and one was home-based. One intervention was conducted at a general practice, and for one study, the setting was unclear. Fifteen studies (58%) were conducted among employees or office workers; this included one study set outside of the worksite. Five (19%) were conducted among older adults, four among the general public (15%), one among parents, and one among a combined sample of school staff and parents.

**Table 1.** Summary of study characteristics.

		**All studies** (26 studies, 38 interventions)	**Studies of worksite-based interventions only** (14 studies, 20 interventions)
Sample size		Combined *N* = 10,355, *N* range: 12–7804, Median *N* = 44	Combined *N* = 1350, *N* range: 12–454, Median *N* = 44
Time to final follow-up		Range: 5 days–12 months Median: 12 weeks	Range: 5 days– 12 months Median: 11 weeks
		Number of studies (% all studies)	Number of studies (% worksite studies)
*Participant descriptions*	Employees/office workers	14 (54%)	14 (100%)
	General public, misc	4 (15%)	*0*
	General public, older adults	5 (19%)	*0*
	Parents	1 (4%)	*0*
	Staff and parents	1 (4%)	*0*
	Office workers and students	1 (4%)	*0*
*Study design*	RCT	15 (58%)	6 (43%)
	Non-RCT	2 (8%)	2 (14%)
	Cluster RCT	2 (8%)	1 (7%)
	Quasi-experiment	3 (12%)	2 (14%)
	Single-arm (pre-post)	4 (15%)	3 (21%)
*Number of arms*	1-arm	4 (15%)	3 (21%)
	2-arm (2 interventions)	9 (35%)	4 (29%)
	2-arm (1 intervention, 1 control)	10 (38%)	5 (36%)
	3-arm (2 interventions, 1 control)	3 (12%)	2 (14%)
*Sedentary behaviour measures (self-reported [SR] or objective [O])*	Waking sedentary time only (O)	2 (8%)	*0*
	Waking sitting time only (O)	1 (4%)	1 (7%)
	Waking sitting time only (SR)	16 (62%)	6 (43%)
	Worksite sitting time only (O)	2 (8%)	2 (14%)
	Worksite sitting time only (SR)	3 (12%)	3 (21%)
	Waking and worksite sedentary time (O)	1 (4%)	1 (7%)
	Waking and worksite sitting time (O)	1 (4%)	1 (7%)
*Theory mentioned*		11 (42%)	2 (14%)
*Sedentary behaviour an eligibility criterion*		6 (23%)	5 (36%)
*Quality score*		Mean = 2.88 (SD = 1.40) Median = 3	Mean = 2.57 (SD = 1.65) Median = 2
		*Number of interventions (% all interventions)*	*Number of interventions (% worksite interventions)*
*Primary behaviour change aim*	To increase physical activity	23 (61%)	11 (55%)
	To reduce sedentary behaviour	8 (21%)	6 (30%)
	Joint: to increase physical activity and reduce sedentary behaviour	2 (5%)	1 (5%)
	Joint: to increase physical activity and improve diet	2 (5%)	0
	To promote weight loss (not behaviour)	2 (5%)	2 (10%)
	Unclear	1 (3%)	0
*Intervention promise*	Very promising	15 (39%)	7 (35%)
	Quite promising	8 (21%)	5 (25%)
	Non promising	15 (39%)	8 (40%)

In total, 10,355 participants were recruited. Sample size ranged from 12 to 7804 (median = 44), though 14 studies (54%) reported samples smaller than 50, and only five (19%) reported sample sizes above 179. Most (22; 85%) were multi-arm trials: 17 randomised controlled trials (RCTs), two non-RCTs, and three quasi-experiments. Thirteen studies (50%) used a no-treatment control group, of which 10 (38% of all studies) were two-arm intervention–control comparisons, and three (12%) compared two interventions with a control. Nine studies (35%) compared multiple (two) interventions only, and four studies (15%) used single-arm designs. Time to final follow-up varied from 5 days to 12 months (median = 12 weeks).

Sedentary behaviour was most commonly measured as self-reported sitting time (19 studies; 73%), but was objectively estimated using combined accelerometer–inclinometers in seven studies (27%), one of which also used direct observation methods. Sedentary behaviour was mostly measured in relation to waking time only (19 studies; 73%), though seven worksite studies assessed worksite-based sedentary behaviour only (5 studies; 16%), or reported separate measures of waking and worksite-based sedentary behaviour (2 studies; 8%).

Eleven studies (42%) mentioned a theory of behaviour, four of which mentioned multiple (two) theories. The theories used were the Transtheoretical Model (seven studies), Social Cognitive Theory (four studies), the Theory of Planned Behaviour (three studies), and Empowerment Theory (one study). Sedentary behaviour was used as an eligibility criterion in six studies (23%). Study quality was generally low (all studies: mean = 2.88, standard deviation [SD] = 1.40, median = 3; RCTs only: mean quality = 3.47, SD = 1.32; non-RCTs, median = 3: mean = 1.78, SD = 0.67, median = 2; Table A3 in Supplemental material).

#### All interventions: intervention characteristics

Of the 38 interventions, 15 (39%) were judged very promising, 8 (21%) quite promising, and 15 (39%) non-promising (Table 2). Eight interventions (21%) primarily aimed to reduce sedentary behaviour, and two (5%) aimed at both increased physical activity and reduced sedentary behaviour, but most (23; 61%) targeted physical activity only. Primary behaviour change aim was related to promise (*χ* ^2^ [2] = 6.20, *p* = .045): very and quite promising interventions primarily targeted sedentary behaviour more often (respectively: 7 of 15 interventions, 46%; 2 of 8 interventions, 25%) than did non-promising interventions (1 of 15 interventions; 7%).

**Table 2.** Intervention characteristics, by intervention promise.

	Interventions				
Characteristics	Very promising (*n* = 15)	Quite promising (*n* = 8)	Non-promising (*n* = 15)	All (*n* = 38)	Promise ratio for intervention functions/behaviour change techniques^a^
***Primary behaviour change aim***					
To reduce sedentary behaviour	5	2	1	8	
Joint: to reduce sedentary behaviour and increase physical activity	2	0	0	2	
To increase physical activity	5	6	12	23	
Other/unclear	3	0	2	5	
***Intervention functions***					** **
**Education**	**4**	**5**	**3**	***12***	***3.0***
**Persuasion**	**1**	**2**	**1**	***4***	***3.0***
Incentivisation	1	0	0	*1*	*–*
**Training**	**3**	**2**	**0**	***5***	***–***
**Environmental restructuring**	**5**	**1**	**0**	***6***	***–***
Modelling	1	0	0	*1*	*–*
Enablement	14	7	12	*33*	*1.8*
***Behaviour change techniques***					*** ***
Goal setting (behaviour)	7	7	9	*23*	*1.6*
**Problem solving**	**4**	**4**	**2**	***10***	***4.0***
**Goal setting (outcome)**	**6**	**0**	**3**	***9***	***2.0***
Action planning	5	5	6	*16*	*1.7*
**Review behavioural goals**	**1**	**3**	**2**	***6***	***2.0***
**Discrepancy between current behaviour and goal**	**2**	**0**	**0**	***2***	***–***
Review outcome goals	1	0	0	*1*	*–*
**Commitment**	**0**	**2**	**0**	***2***	***–***
Monitoring behaviour by others without feedback	1	0	1	*2*	*1.0*
**Feedback on behaviour**	**7**	**1**	**4**	***12***	***2.0***
Feedback on outcomes	0	1	0	*1*	*–*
**Self-monitoring (behaviour)**	**8**	**4**	**3**	***15***	***4.0***
Self-monitoring (outcome)	1	0	3	*4*	*0.5*
Biofeedback	0	0	1	*1*	*–*
**Social support (unspecified)**	**11**	**5**	**7**	***23***	***2.3***
**Social support (practical)**	**2**	**0**	**1**	***3***	***2.0***
Instruction on how to perform behaviour	7	4	7	*18*	*1.6*
**Information on health consequences**	**4**	**4**	**3**	***11***	***2.7***
Information on social and emotional consequences	1	0	1	*2*	*1.0*
Social comparison	1	1	0	*2*	*–*
**Prompts/cues**	**2**	**2**	**2**	***6***	***2.0***
**Behavioural practice/rehearsal**	**3**	**1**	**0**	***4***	***–***
**Behaviour substitution**	**3**	**2**	**2**	***7***	***2.5***
**Habit formation**	**1**	**1**	**0**	***2***	***–***
Habit reversal	0	1	0	*1*	*–*
Graded tasks	4	2	5	*11*	*1.2*
Credible source	1	1	2	*4*	*1.0*
**Pros and cons**	**3**	**1**	**0**	***4***	***–***
Material incentive for behaviour	1	0	0	*1*	* *
Material reward for behaviour	1	0	0	*1*	* *
**Social reward**	**2**	**1**	**0**	***3***	***–***
Self-reward	0	1	0	*1*	*–*
**Restructuring the physical environment**	**4**	**1**	**0**	***5***	***–***
**Restructuring the social environment**	**3**	**0**	**1**	***4***	***3.0***
Adding objects to the environment	11	1	7	*19*	*1.7*
Identification of self as role model	1	0	0	*1*	*–*
Verbal persuasion about capability	0	0	1	*1*	*–*

^a^Promise ratio denotes the number of very or quite-promising interventions in which an intervention function or behaviour change technique featured, divided by the number of non-promising interventions in which it featured. Promise ratios only calculable for functions or techniques used in both promising and non-promising interventions. Rows in bold denote functions or techniques associated with a promise ratio of 2 or above, or used exclusively in promising interventions and featuring in at least two interventions.

##### Intervention functions

Seven intervention functions were each observed in at least one intervention. Intervention promise was linked to the number of functions (*F* [2,35] = 3.86, *p* = .03): very promising (mean functions per intervention = 1.93, SD = 1.28) and quite promising interventions (mean functions = 2.13, SD = 1.13) reported more functions than did non-promising interventions (mean functions = 1.07, SD = 0.59; *t* [18.93] = 5.54, *p* < .001). Quite promising interventions reported more functions than did non-promising interventions (*t* [9.13] = 2.48, *p* = .04).

Most frequently used were enablement (33 interventions; 87%), education (12 interventions; 32%), and environmental restructuring (6 interventions; 16%). Four intervention functions were deemed promising: education (promise ratio = 3.0), persuasion (ratio = 3.0), environmental restructuring (6 interventions, all of which were promising), and training (5 interventions, all promising).

##### Behaviour change techniques

Thirty-seven behaviour change techniques were each used in at least one intervention. There was no overall association between intervention promise and the number of techniques (*F*[2,35] = 1.59, *p* = .22), but promising interventions used more techniques (very promising, mean number of techniques per intervention = 7.27, SD = 5.19; quite promising, mean techniques = 7.00, SD = 2.83) than did those with no evidence of promise (mean techniques = 4.87, SD = 2.70; *t* [27.59] = 5.19, *p* < .001). Quite promising and non-promising interventions did not differ (*t*[13.81] = 1.75, *p* = .10).

The most frequently observed behaviour change techniques were setting behavioural goals (23 interventions; 61%), providing unspecified forms of social support (23 interventions; 61%), and adding objects to the environment (19 interventions; 50%). Eighteen techniques were found to be promising, for eleven of which promise ratios could be calculated: self-monitoring behaviour (used in 15 interventions; promise ratio = 4.0), problem solving (10 interventions, ratio = 4.0), restructuring the social environment (4 interventions, ratio = 3.0), providing information on health consequences (11 interventions, ratio = 2.7), behaviour substitution (7 interventions, ratio = 2.5), unspecified social support (ratio = 2.3), providing feedback on behaviour (12 interventions, ratio = 2.0), setting outcome goals (9 interventions, ratio = 2.0), reviewing behavioural goals (6 interventions, ratios = 2.0), using prompts or cues (6 interventions, ratio = 2.0), and providing practical social support (3 interventions, ratio = 2.0). Restructuring the physical environment (5 interventions), behavioural practice or rehearsal (4 interventions), pros and cons (4 interventions), social rewards (3 interventions), and habit formation, commitment, and discrepancy between current behaviour and goals (2 interventions respectively) were used in promising interventions only.

#### Worksite-based interventions only: intervention characteristics

Of the twenty interventions, seven (35%) were judged very promising, five (25%) quite promising, and eight (40%) non-promising. Primary behaviour change aim was related to promise (*χ* ^2^ [2] = 6.36, *p* = .02): very promising interventions tended to have primarily targeted sedentary behaviour either solely or jointly with physical activity (5 of 7 interventions; 71%), but quite promising (1 of 5; 20%) and non-promising interventions (1 of 8; 13%) did not.

##### Intervention functions

Intervention promise was linked to the number of functions (*F* [2,17] = 4.38, *p* = .03): very promising (mean functions per intervention = 2.43, SD = 1.62) and quite promising interventions (mean functions = 1.40, SD = 0.55) used more functions than did non-promising interventions (mean functions = 0.88, SD = 0.35; *t*[8.34] = 4.20, *p* = .002). Quite and non-promising interventions did not differ (*t*[6.12] = 1.91, *p* = .10).

Most frequently used were enablement (16 interventions; 80%) and environmental restructuring (6; 30%), the latter mostly capturing provision of standing workstations (5; 25%; Table A4 in Supplemental material). Two functions were deemed promising: environmental restructuring (used in promising interventions only), and education (5 interventions; promise ratio = 4.0).

##### Behaviour change techniques

Twenty-eight behaviour change techniques were each observed in at least one intervention. There was no overall association between intervention promise and the number of techniques (*F*[2,17] = 2.02, *p* = .16), but more promising interventions used more techniques (very promising, mean number of techniques per intervention = 8.57, SD = 6.78; quite promising, mean techniques = 5.60, SD = 2.07) than did non-promising interventions (mean techniques = 4.13, SD = 1.81; *t*[8.30] = 3.59, *p* = .007). Quite promising and non-promising interventions did not differ (*t*[7.71] = 1.31, *p* = .23).

The most frequently observed techniques were setting behavioural goals (13 interventions; 65%), providing unspecified forms of social support (10 interventions; 50%), instructing on how to perform the behaviour (10 interventions; 50%), self-monitoring behaviour (9; 45%), adding objects to the environment (9; 45%), and action planning (8; 40%). Fourteen techniques were found to be promising, for six of which promise ratios could be calculated: self-monitoring of behaviour (promise ratio = 3.5), adding objects to the environment (ratio = 3.5), instruction on how to perform the behaviour (ratio = 2.3), reviewing behavioural goals, providing information on health consequences, and behaviour substitution (each used in 6 interventions, ratios = 2.0). Restructuring the physical environment (used in 5 interventions), problem solving (4 interventions), discrepancy between current behaviour and goal (2 interventions), feedback on behaviour (2 interventions), providing practical social support (2 interventions), social comparison (2 interventions), behavioural practice or rehearsal (2 interventions), and restructuring the social environment (2 interventions) were used in promising interventions only.

## Discussion

This review is the first to have focused on the behaviour change methods used in sedentary behaviour change interventions in adults. Twenty-six studies, reporting 38 interventions, were identified. Despite studies generally being low-quality, and intervention design mostly eschewing behavioural theory, the interventions generally showed promise in reducing sedentary behaviour. Four intervention functions – education, environmental restructuring, persuasion, and training – showed potential for engineering reduction in sedentary behaviour. Eighteen behaviour change techniques showed promise, with self-monitoring of behaviour, problem solving, and changing the social or physical environment showing particular promise.

Sedentary behaviour has traditionally been equated with physical inactivity, and many interventions in our review reported sedentary behaviour as one of many outcomes related to physical activity. The most promising interventions were those that primarily aimed to change sedentary behaviour, rather than physical activity. Several interventions focusing on physical activity that were included in this review achieved changes in activity, but not sedentary behaviour. While time spent in sedentary behaviour tends to correlate negatively with time in moderate-to-vigorous activity (Mansoubi, Pearson, Biddle, & Clemes, 2014; Ryan et al., 2011), this relationship is often small to moderate in strength. As our data suggest, reduction of sedentary behaviour is not an inevitable consequence of effective activity-promotion interventions. This demonstrates the importance of treating sedentary behaviour as independent of moderate-to-vigorous physical activity when designing interventions to reduce sedentary time (Prince et al., 2014).

One intervention that targeted sedentary behaviour only had no impact on sedentary time (Evans et al., 2012). This highlights the potential for sedentary behaviour change interventions to fail, and testifies to the importance of identifying intervention components that may contribute to effectiveness (Michie & Abraham, 2004). We applied two coding frames to characterise interventions according to the functions that they played (e.g., education, persuasion, training), and the behaviour change techniques used. Using these coding frames to analyse existing interventions can reveal the approaches and techniques associated with effective interventions (Dombrowski et al., 2012; Gilinsky et al., 2015; Martin et al., 2013). Our review showed that self-monitoring behaviour, problem solving, modifying social and physical environments, and giving information on the health impact of sitting were most closely associated with promising interventions. This echoes previous work identifying provision of sit-stand desks and personalised advice as effective in reducing sedentary behaviour (Shrestha et al., 2015). The techniques identified here might fruitfully be incorporated into future sedentary behaviour change interventions.

Where explicit theory use is rare, as was found among many interventions in this review, identifying common intervention functions and techniques can also reveal the implicit assumptions that intervention developers have made regarding why people engage in sedentary behaviour and do not spend more time standing or in light or moderate-to-vigorous physical activity (see Gardner et al., 2010). The most frequently used intervention functions were enablement (i.e., facilitating reduction in sedentary behaviour), education, and environmental restructuring, and the most commonly used techniques were setting behavioural goals, providing unspecified forms of social support, and adding objects to the environment (activity monitors or sit-stand desks). This suggests that intervention developers have tended to conceive of sedentary behaviour as largely determined by external environments, or as a self-regulatory problem, and that people would be willing to reduce their sedentary time if the environment were modified, or if supported in developing self-regulatory skills for sitting less. These assumptions are not unfounded; modifying the environment by providing sit-stand desks often reduces time spent sedentary (e.g., Alkhajah et al., 2012), and self-regulatory skills, such as self-monitoring, action planning, and goal setting, have been shown to be potent techniques across behaviour domains, including increasing moderate-to-vigorous physical activity (Dombrowski et al., 2012; Gilinsky et al., 2015; Michie, Abraham, et al., 2009). Yet, behaviour is determined by capability, opportunity, and motivation (Michie et al., 2011). By focusing primarily on increasing individuals’ psychological capability to reduce sedentary behaviour (through, for example, goal setting), and maximising opportunities to limit or restrict sedentary behaviour (e.g., through modifying the physical environment). Sedentary behaviour reduction interventions may have given insufficient attention to motivation. Surprisingly few interventions sought to motivate participants through information provision or education. The dangers of sedentary behaviour may be poorly understood, given that it has only relatively recently been recognised as a health concern within the scientific community (SBRN, 2012). Future work might investigate the scope to bring about significant population-level reductions in sedentary behaviour through information provision.

Our results highlight a reliance to date on study designs from which definitive conclusions about intervention effectiveness cannot be drawn. Only six studies specified sedentary behaviour as an inclusion criterion. Intervention effects may be underestimated where the most sedentary, who have most to benefit, are not especially targeted. The observed effectiveness of any intervention is also dependent on the nature of the control group; effects may be suppressed where those in the comparison condition also receive an intervention (de Bruin, Viechtbauer, Hospers, Schaalma, & Kok, 2009). True estimation of the effectiveness of sedentary behaviour reduction interventions requires comparison with a group that received no treatment. Yet, only half of all studies employed RCT designs with no-treatment control groups. Several studies used uncontrolled study designs, which are particularly problematic. One study indicated that, among an older adult sample, TV viewing – i.e., a behaviour commonly undertaken while sedentary – increased over time (Gardner, Iliffe, Fox, Jefferis, & Hamer, 2014). An apparent lack of change in sedentary behaviour within an uncontrolled study may represent significant behaviour change relative to a control group for whom sedentary behaviour naturally increased. We recognise the utility of uncontrolled designs for cost-effective, early-phase intervention piloting (Fitzsimons et al., 2013). Nonetheless, the evidence base for ‘what works’ and ‘why’ with regards to reducing sedentary behaviour is presently weak due to suboptimal evaluation designs.

Limitations of our study must be acknowledged. The evidence synthesis was undertaken to point to which strategies offer promise for reducing sedentary behaviour. Our classification system, whereby the level of intervention promise was determined based on whether between- or within-group change was observed on at least one measure of sedentary behaviour, was ultimately arbitrary. We may also have overestimated the true potential of two interventions for which change was observed on only one of multiple sedentary behaviour indices (Alkhajah et al., 2012; Evans et al., 2012). Our judgements of intervention potential cannot be directly compared with those from previous reviews of the effectiveness of sedentary behaviour change interventions, which used standardised outcomes (Prince et al., 2014; Shrestha et al., 2015). Intervention promise was partly determined by between-group change, so may have been underestimated in five studies that assessed multiple, equally effective interventions in the absence of a no-treatment control arm.

Studies were included only where sedentary behaviour was measured objectively, using an accelerometer–inclinometer, which reliably distinguishes sitting from standing or light activity (Grant et al., 2006), or as self-reported time spent sitting. These criteria excluded studies in which sedentary behaviour was inferred from accelerometry data indicating minimal or no physical activity, but retained studies using self-report measures, which can underestimate true sedentary behaviour (Aguilar-Farías, Brown, Olds, & Peeters, 2014). Physical activity and sedentary behaviour are, however, conceptually discrete (SBRN, 2012), and are thought to pose independent health risks (Wilmot et al., 2012), and so syntheses of sedentary behaviour change interventions demand outcome measures that discern sedentary behaviour from physical activity. A comparative study showed that accelerometer–inclinometers yielded near-perfect correlation with directly observed sedentary minutes (*r* = 0.94), whereas an accelerometer without inclinometer produced a considerably weaker correlation (*r* = 0.39; Kozey-Keadle, Libertine, Lyden, Staudenmayer, & Freedson, 2011). Additionally, our self-report criteria excluded studies in which sedentary behaviour was inferred from time spent in typically sedentary tasks, such as screen time (TV viewing, computer use), reading, talking on the phone, or engaging in hobbies (Gardiner, Clark, et al., 2011). Our search identified five studies that had sought to reduce time spent in these activities, which would otherwise have met our inclusion criteria (Fitzgibbon et al., 2005; French, Gerlach, Mitchell, Hannan, & Welsh, 2011; Jago et al., 2013; Otten, Jones, Littenberg, & Harvey-Berino, 2009; Steeves, Bassett, Fitzhugh, Raynor, & Thompson, 2012). The five studies reported seven interventions. All focused on TV-viewing time, of which one was very promising, three quite promising, and two non-promising, while for one, promise could not be reliably coded because statistical significance of observed changes was not reported (Jago et al., 2013). (For completeness, the characteristics of these studies, including intervention functions and behaviour change techniques, are provided in Table A5 in Supplemental material.) While exclusion of these studies may have overlooked important strategies for reducing sedentary screen time, time spent in typically sedentary tasks does not reliably reveal sedentary behaviour. Studies of the determinants of sedentary behaviour have treated TV-viewing as an archetypal sitting-based activity (Rhodes et al., 2012), and a study (published after our review was completed) suggested that, on average, seated TV-viewing yields an energy expenditure of 1.33 METs/min (Mansoubi et al., 2015), so meets the definition of a sedentary behaviour (SBRN, 2012). Yet, this estimate was based on participants who were asked to watch TV while seated. To our knowledge, there is no evidence available on the extent to which – or the proportion of people for whom – real-world TV-viewing is a truly sedentary activity. Moreover, using TV-viewing as an indicator of sedentary behaviour in intervention research neglects the possibility that, by incorporating light activity into otherwise sedentary routines (Rovniak et al., 2014; Steeves et al., 2012), substantial reductions may be achieved in sedentary behaviour with little or no change in viewing time. The development of effective strategies to reduce sedentary behaviour depends upon intervention evaluations using measures that distinguish true sedentary behaviour from time spent in activities that are normally performed while seated, but could be performed while physically active.

We judged intervention components to have potential where they were used in two or more interventions, and at least twice as many promising as non-promising interventions. These criteria may have been too conservative, neglecting potentially fruitful components that may have been infrequently used. Interventions and components deemed ‘non-promising’ may not be ineffective; rather, there is presently insufficient or inconsistent evidence for them to be recommended. Publication bias makes possible that the potential contributions of some intervention functions and techniques to reductions in sedentary behaviour may have been overstated because ineffective interventions featuring such components were unavailable for review. Exploratory studies might test more rigorously the effectiveness of lesser-used functions and techniques, or those for which evidence is inconclusive.

Interventions are typically poorly reported (Lorencatto, West, Stavri, & Michie, 2013), and so potentially important intervention components may not have been coded. Some techniques and functions may be more likely to be reported than others (Lorencatto et al., 2013). While we approached authors for further information, the response rate was low. This emphasises the need for thorough intervention descriptions to be made publicly accessible. We echo previous calls for interventions to be described using the standardised terminology of the Behaviour Change Technique Taxonomy v1 (Michie et al., 2013) and for additional materials to be posted as supplementary material or made permanently available in institutional repositories.

Our coding approach decontextualised intervention components. We coded for the presence of techniques, but not the frequency or intensity with which they were used. Intervention reports do not often describe the frequency with which techniques are delivered; extracting such information typically requires observation of intervention delivery sessions (Lorencatto, West, Christopherson, & Michie, 2013). Moreover, we coded for functions and techniques used to target either sedentary behaviour or physical activity, and it is possible that intervention components that successfully increase physical activity may not effectively reduce sedentary behaviour, and vice versa. However, it is difficult to conceptually separate physical activity promotion and sedentary behaviour reduction components: some interventions apparently targeted activity only, yet achieved changes in sedentary behaviour, whereas others targeted both activity and sedentary behaviour, and either sought to substitute sedentary behaviour for physical activity, or failed to distinguish potentially discrete activity- and sedentary-focused components. Future evidence syntheses will be aided by clearer delineation, where possible, of strategies used to change physical activity and sedentary behaviour, respectively, in intervention reports. Relatedly, because we did not code to which behaviour each intervention component related, some techniques may have appeared unpromising because they were used in relation to increasing physical activity (e.g., self-monitoring bouts of activity), rather than reducing sedentary behaviour (self-monitoring sitting time). Additionally, techniques can have greater effect where delivered as part of a package, rather than in isolation. Previous work has shown that combining self-regulatory behaviour change techniques yields greater effects than any of these techniques in isolation (Dombrowski et al., 2012; Michie, Abraham, et al., 2009). More broadly, intervention effectiveness may depend not only on functions and BCTs, but also on how they are delivered, to whom, in that format, with what intensity, and for how long (Davidson et al., 2003). These elements were outside of the scope of this review, but nonetheless, interactions between content and other characteristics not coded here may perhaps explain variation. It is consequently unclear whether the functions or techniques identified here as promising would retain their promise across all settings and populations. Indeed, our supplementary analyses showed different patterns of promising functions and techniques across worksite and non-worksite settings. Worksite sedentary behaviour may be more receptive to, for example, prior planning and routinisation than non-worksite sedentary behaviour, which occurs in less predictable and structured contexts. Intervention components should be chosen on the basis of what is most appropriate and feasible in the local setting (Michie, Atkins, & West, 2014).

Behavioural interventions show promise for reducing sedentary behaviour, and those designed with the primary aim of reducing sedentary behaviour rather than increasing physical activity show the most promise. Intervention designers should consider using environmental restructuring, and self-regulatory techniques such as self-monitoring, problem solving, and providing information on health consequences, as these have been more common in promising than non-promising interventions. Yet, the evidence base is weakened by low-quality study designs, reliance on self-report, and small samples. Future trials should employ more rigorous evaluation methods, ideally using accelerometer–inclinometer data from large RCTs with no-treatment comparison groups.

## Note

1. Our adapted tool differs from that used by Chau et al. (2010) on only one item, on which Chau et al. coded for whether point estimates and validity were reported for the primary outcome of the original study, whereas we coded for point estimates and validity in relation to the sedentary behaviour index used as a measure of intervention potential in our analyses.
