# Supplementary material for: How to reduce sitting time? A review of behaviour change strategies used in sedentary behaviour reduction interventions among adults
Source: Health Psychol Rev. 2015 Sep 16;10(1):89–112. doi: 10.1080/17437199.2015.1082146 (PMC4743603; doi:10.1080/17437199.2015.1082146)
Supplement: Supplemental References.pdf [file rhpr_a_1082146_sm1781.pdf]

**Supplemental references**

** References marked with an asterisk were retrieved by the systematic search and included in the review. ** References marked with two asterisks provided additional description of one or more intervention treatments.*

* Aittasalo, M., Miilunpalo, S., & Suni, J. (2004) The effectiveness of physical activity counseling in a work-site setting. A randomized, controlled trial. *Patient Education and Counseling, 55,* 193-202. doi: 10.1016/j.pec.2003.09.003.

** Aittasalo, M., & Miilunpalo, S., (2006) Offering physical activity counselling in occupational health care – does it reach the target group? *Occupational Medicine, 56,* 55-58. doi: :10.1093/occmed/kqi191.

* Alkhajah, T.A., Reeves, M.M., Eakin, E.G., Winkler, E.A.H., Owen, N., & Healy, G.N. (2012) Sit-stand workstations. A pilot intervention to reduce office sitting time. *American Journal of Preventive Medicine, 43,* 298-303. doi: 10.1016/j.amepre.2012.05.027.

** Baker, G., Gray, S.R., Wright, A., Fitzsimons, C., Nimmo, M., Lowry, R., & Mutrie, N. (2008) The effect of a pedometer-based community walking intervention "Walking for Wellbeing in the West" on physical activity levels and health outcomes: a 12-week randomized controlled trial. *International Journal of Behavioral Nutrition and Physical Activity, 5***,** 44. doi: 10.1186/1479-5868-5-44.

* Barwais, F.A, Cuddihy, T.F., & Tomson, L.M. (2013) Physical activity, sedentary behavior and total wellness changes among sedentary adults: a 4-week randomized controlled trial. *Health and Quality of Life Outcomes, 11,* 183. doi: 10.1186/1477-7525-11-183.

* Burke, L., Lee, A.H., Jancey, J., Xiang, L., Kerr, D.A., Howat, P.A., Hills, A.P., & Anderson, A.S. (2013) Physical activity and nutrition behavioural outcomes of a home-based intervention program for seniors: a randomized controlled trial. *International Journal of Behavioral Nutrition and Physical Activity, 10,* 14. doi: 10.1186/1479-5868-10-14..

** Burke, L., Jancey, J., Howat, P., Lee, A., Kerr, D., Shilton, T., Hills, A., Anderson, A. (2010) Physical activity and nutrition program for seniors (PANS): protocol of a randomized controlled trial. *BMC Public Health, 10,* 751. doi: 10.1186/1471-2458-10-751.

* Chang, A.K., Fritschi, C., Kim, M.J. (2013) Sedentary behavior, physical activity, and psychological health of Korean older adults with hypertension. Effect of an empowerment intervention. *Research in Gerontological Nursing, 6,* 81-88. doi: 10.3928/19404921-20121219-01.

* De Cocker, K., Spittaels, H., Cardon, G., De Bourdeaudhuij, I., & Vandelanotte, C. (2012) Web-based, computer-tailored, pedometer-based physical activity advice: Development, dissemination through general practice, acceptability, and preliminary efficacy in a randomized controlled trial. *Journal of Medical Internet Research, 14,* e53. doi: 10.2196/jmir.1959.

* Dewa, C.S., deRuiter, W., Chau, N., & Karioja, K. (2009) Walking for Wellness: Using pedometers to decrease sedentary behaviour and promote mental health. *International Journal of Mental Health Promotion, 11,* 24-28. doi: 10.1080/14623730.2009.9721784.

* Dunn, A.L., Garcia, M.E., Marcus, B.H., Kamper, J.B., Kohl, H.W., & Clair, S.N. (1998) Six-month physical activity and fitness changes in Project Active, a randomized trial. *Medicine & Science in Sports and Exercise, 30,* 1076-1083.

** Dunn, A.L., Marcus, B.H., Kampert, J.B., Garcia, M.E., Kohl, H.W., Blair, S.N. (1997) Reduction in cardiovascular disease risk factors: six-month results from Project Active. *Preventive Medicine, 26,* 883-892.

** Dunstan, D.W., Wiesner, G., Eakin, E.G., Neuhaus, M., Owen, N., LaMontagne, A.D., Moodie, M., Winkler, E.A.H., Fjeldsoe, B.S., Lawler, S., & Healy, G.N. (2013) Reducing office workers' sitting time: rationale and study design for the Stand Up Victoria cluster randomized trial. *BMC Public Health, 13,* 1057. doi: 10.1186/1471-2458-13-1057.

* Ellegast, R., Weber, B., & Mahlberg, R (2012) Method inventory for assessment of physical activity at VDU workplaces. *Work, 41,* 2355-2359. doi: 10.3233/WOR-2012-0464-2355.

* Evans, R.E., Fawole, H.O., Sheriff, S.A., Dall, P.M., Grant, P.M., & Ryan, C.G. (2012) Point-of-choice prompts to reduce sitting time at work. A randomized trial. *American Journal of Preventive Medicine, 43,* 293-297. doi: 10.1016/j.amepre.2012.05.010.

** Fitzsimons, C.F., Baker, G., Wright, A., Nimmo, M.A., Ward Thompson, C., Lowry, R., Millington, C., Shaw, R., Fenwick, E., Ogilvie, D., Inchley, J., Foster, C.E., & Mutrie, N (2008) The ‘Walking for Wellbeing in the West’ randomised controlled trial of a pedometer-based walking programme in combination with physical activity consultation with 12 month follow-up: rationale and study design. *BMC Public Health, 8,* 259. doi: 10.1186/1471-2458-8-259.

* Fitzsimons, C.F., Baker, G., Gray, S.R., Nimmo, M.A., Mutrie, N. (2012) Does physical activity counseling enhance the effects of a pedometer-based intervention over the long-term: 12-month findings from the Walking for Wellbeing in the West study. *BMC Public Health, 12,* 206. doi: 10.1186/1471-2458-12-206.

* Fitzsimons, C.F., Kirk, A., Baker, G., Michie, F., Kane, C., & Mutrie, N. (2013) Using an individualised consultation and activPAL feedback to reduce sedentary time in older Scottish adults: Results of a feasibility and pilot study. *Preventive Medicine, 57,* 718-720. doi: 10.1016/j.ypmed.2013.07.017.

* Gilson, N.D., Puig-Ribera, A., McKenna, J., Brown, W.J., Burton, N.W., & Cooke, C.B. (2009) Do walking strategies to increase physical activity reduce reported sitting in workplaces: a randomized control trial. *International Journal of Behavioral Nutrition and Physical Activity, 6,* 43. doi: 10.1186/1479-5868-6-43.

* Hansen, A.W., Grønbæk, M., Wulff Helge, J., Severin, M., Curtis, T., Schurmann Tolstrup, J. (2012) Effect of a web-based intervention to promote physical activity and improve health

among physically inactive adults: A population-based randomized controlled trial. *Journal of Medical Internet Research, 14,* e145. doi: 10.2196/jmir.2109.

* Healy, G.N., Eakin, E.G., LaMontagne, A.D., Owen, N., Winkler, E.A.H., Wiesner, G., Gunning, L., Neuhaus, M., Lawler, S., Fjeldsoe, B.S., & Dunstan, D.W. (2013) Reducing sitting time in office workers: Short-term efficacy of a multicomponent intervention. *Preventive Medicine, 57,* 43-48. doi: 10.1016/j.ypmed.2013.04.004.

* John, D., Thompson, D.L., Raynor, H., Bielak, K., Rider, B., Bassett, D.R. (2011) Treadmill workstations: A worksite physical activity intervention in overweight and obese office workers. *Journal of Physical Activity and Health, 8,* 1034-1043.

** Kohl, H.W., Dunn, A.L., Marcus, B.H., Blair, S.N. (1998) A randomized trial of physical activity interventions: design and baseline data from Project Active. *Medicine & Science in Sports and Exercise, 30,* 275-283.

* Kozey-Keadle, S., Libertine, A., Lyden, K., Staudenmayer, J., Freedson, P.S. (2011) Validation of wearable monitors for assessing sedentary behavior. *Medicine & Science in Sports and Exercise, 43,* 1561-1567. doi: 10.1249/MSS.0b013e31820ce174.

** MacMillan, F., Fitzsimons, C., Black, K., Granat, M.J., Grant, M.P., Grealy, M., Macdonald, H., McConnachie, A., Rowe, D.A., Shaw, R., Skelton, D.A., & Mutrie, N (2011) West End Walkers 65+: A randomised controlled trial of a primary care-based walking intervention for older adults: Study rationale and design. *BMC Public Health, 11,* 120. doi: 10.1186/1471-2458-11-120.

* Marshall, A.L., Leslie, E.R., Bauman, A.E., Marcus, B.H., & Owen, N. (2003a) Print versus website physical activity programs. A randomized trial. *American Journal of Preventive Medicine, 25,* 88-94. doi: 10.1016/S0749-3797(03)00111-9.

** Marshall, A.L., Bauman, A.E., Owen, N., Booth, M.L., Crawford, D., & Marcus, B.H. (2003b) Population-based randomized controlled trial of a stage-targeted physical activity intervention. *Annals of Behavioral Medicine, 25,* 194-202.

* Mazzeo, S.E., Gow, R.W., Stern, M., & Gerke, C.K. (2008) Developing an intervention for parents of overweight children. *International Journal of Child and Adolescent Health, 1,* 355-363.

* Mutrie, N., Doolin, O., Fitzsimons, C.F., Grant, P.M., Granat, M., Grealy, M., Macdonald, H., MacMillan, F., McConnachie, A., Rowe, D.A., Shaw, R., & Skelton, D.A. (2012) Increasing older adults' walking through primary care: results of a pilot randomized controlled trial. *Family Practice, 29*, 633-642. doi: 10.1093/fampra/cms038.

** Neuhaus, M., Healy, G.N., Fjeldsoe, B.S., Lawler, S., Owen, N., Dunstan, D.W., LaMontagne, A.D., & Eakin, E.G. (2014) Iterative development of Stand Up Australia: a multi-component intervention to reduce workplace sitting. *International Journal of Behavioral Nutrition and Physical Activity, 11,* 21. doi: 10.1186/1479-5868-11-21.

* Opdenacker, J., & Boen, F. (2008) Effectiveness of face-to-face versus telephone support in increasing physical activity and mental health among university employees. *Journal of Physical Activity & Health, 5,* 830-843.

* Østeras, H., & Hammer, S. (2006) The effectiveness of a pragmatic worksite physical activity program on maximal oxygen consumption and the physical activity level in healthy people. *Journal of Bodywork and Movement Therapies, 10,* 51-57. doi: 10.1016/j.jbmt.2005.02.003.

* Pronk, N. P., Katz, A. S., Lowry, M., & Payfer, J. R. (2012). Reducing occupational sitting time and improving worker health: The Take-a-Stand Project, 2011. *Preventing Chronic Disease, 9,* 110323. doi: 110310.115888.pcd110329.110323.

* Shaw, K.L., & Page, C. (2008) A pilot community-based walking-for-exercise program for senior women. *Topics in Geriatric Rehabilitation, 24,* 315-324. doi: 10.1097/TGR.0b013e31818cd005.

** Spittaels, H., & De Bourdeaudhuij, I. (2007) Who participates in a computer-tailored physical activity program delivered through the Internet? A comparison of participants' and

non-participants' characteristics. *International Journal of Behavioral Nutrition and Physical Activity, 4*, 39. doi: 10.1186/1479-5868-4-39.

* Spittaels, H., De Bourdeaudhuij, I., & Vandelanotte, C. (2007) Evaluation of a website-delivered computer-tailored intervention for increasing physical activity in the general population. *Preventive Medicine, 44,* 209-217. doi: 10.1016/j.ypmed.2006.11.010.

* Verweij, L.M., Proper, K.I., Weel, A.N.H., Hulshof, C.T.J., & van Mechelen, W. (2012) The application of an occupational health guideline reduces sedentary behaviour and increases fruit intake at work: results from an RCT. *Occupational and Environmental Medicine, 69,* 500-507. doi: 10.1136/oemed-2011-100377.
