# Supplementary material for: How to reduce sitting time? A review of behaviour change strategies used in sedentary behaviour reduction interventions among adults
Source: Health Psychol Rev. 2015 Sep 16;10(1):89–112. doi: 10.1080/17437199.2015.1082146 (PMC4743603; doi:10.1080/17437199.2015.1082146)
Supplement: Supplemental Table_1.pdf [file rhpr_a_1082146_sm1780.pdf]

Supplemental Table 1. Search strategy developed in Ovid, used to concurrently search Embase, Medline, PsycInfo, and PsycArticles

Search step	*Search term*	*Fields searched*	*Comments/justification of search at each step*
#1	(reduc* or decreas* or discourag* or lessen* or chang* or modif* or alter* or improv*) adj2 (sitting or seated or sedentary or inactiv* or TV or television or screen or internet or computer)	Title OR abstract OR keywords	Focuses on reducing sedentary behaviour
#2	(promot* or uptake or encourag* or increas* or start or chang* or modif* or alter* or improv*) adj ((physical* adj activ*) or exercis* or walk* or fitness or sport or stand*)	Title OR abstract OR keywords	Focuses on increasing physical activity
#3	#1 OR #2		Combines steps 1 and 2: reduction of sedentary behaviour or increase in physical activity
#4	(sitting or seated or sedentary or inactiv* or TV or television or screen or internet or computer)	Title OR abstract OR keywords	Focuses on sedentary behaviour
#5	#3 AND #4		Combines steps 3 and 4: ensures studies on decreasing sedentary behaviour or increasing physical activity include a measure of sedentary behaviour
#6	(random* adj control* adj (trial OR study)) OR (clinical adj trial) OR (compar* adj (group OR study OR trial)) OR (control* adj (group OR study OR trial)) OR (match* adj (group OR study OR trial)) OR (random* adj assign*)	Title OR abstract OR keywords OR methodology	Focuses on intervention evaluation study designs
#7	(adult* not child* not teenage* not adolescen* NOT (strok* OR pregnan* OR osteoporo* OR COPD OR (chronic adj obstructive) OR asthma OR (back adj pain) OR angina OR (heart adj disease) OR (heart adj failure) OR cancer OR psychiatr* OR (cerebral adj palsy) OR arthrit* OR rheumat* OR fibromyalgi* OR (nursing adj home) OR rehabilitat*))	Title OR abstract OR keywords OR methodology OR age group OR population group	Focuses on ineligible participant groups

#8	#5 AND #6 AND #7		Combines steps 5-7: specifies sedentary behaviour interventions, using intervention trial designs, excluding ineligible participants
#9	Limit to: English language, human		Limits results to publications in English, among humans
#10	Remove duplicates		Deduplicates results
