# Supplementary material for: How to reduce sitting time? A review of behaviour change strategies used in sedentary behaviour reduction interventions among adults
Source: Health Psychol Rev. 2015 Sep 16;10(1):89–112. doi: 10.1080/17437199.2015.1082146 (PMC4743603; doi:10.1080/17437199.2015.1082146)
Supplement: Supplemental Table_2.pdf [file rhpr_a_1082146_sm1778.pdf]

Supplemental Table 2. Description of studies

Reference and misc details	*Sedentary behaviour measures **	*Quality*	*Description of intervention treatment(s)*	*Intervention group(s) description*	*Control group description*	*Intervention content*	*Promise*
Aittasalo et al (2004) (also Aittasalo & Miilunpalo, 2006) ‡<br><br>Finland<br><br>3-arm RCT (2 interventions, 1 control)<br><br>Follow-up**: 12 months only<br><br>Theory mentioned? Yes (Transtheoretical Model)<br><br>Sample: Employees	Waking sitting time on workday *(unclear whether typical or actual workday)* (SR)<br><br>Waking sitting time on a non-workday *(unclear whether typical or actual)* (SR) *	3/7	<u>Intervention 1:</u> behavioural counseling promoting PA<br><br><u>Intervention 2:</u> behavioural counseling promoting PA + fitness testing	<u>Intervention 1</u> (counseling only)<br><br>N = 52<br>Mean age 45y<br>61% female<br><br><u>Intervention 2</u> (counseling + fitness)<br><br>N = 51<br>Mean age 44y<br>53% female	N = 52<br>Mean age 42y<br>54% female	<u>Intervention 1:</u><br>Behaviour types targeted: PA<br><br>Primary behaviour change aim: to increase PA<br><br>Functions: Enablement<br><br>BCTs: Goal setting (behaviour); Action planning; Review behaviour goals; Social support (unspecified); Information about health consequences<br><br><u>Intervention 2:</u><br>Behaviour types targeted: PA<br><br>Primary behaviour change aim: to increase PA<br><br>Functions:<br>Enablement, education<br><br>BCTs: Goal setting (behaviour); Action planning; Review	<u>Intervention 1:</u><br>**Non-promising**<br>*Within-group:*<br>Not reported.<br><br>*Between-group:*<br>Smaller change in non-workday sitting relative to Intervention 2. Comparison with control not reported.<br><br><u>Intervention 2:</u><br>**Quite promising**<br><br>*Within-group:*<br>Not reported.<br><br>*Between-group:*<br>Greater reduction in non-workday sitting relative to Intervention 1 or control group. No

SB an explicit eligibility criterion?<br>No<br><br>Setting: Worksite<br><br>Total sample at follow-up*:<br>N = 155						behaviour goals; Feedback on outcome of behaviour; Social support (unspecified); Information about health consequences; Social comparison	effects on other sitting indices.
Alkhajah et al (2012)<br><br>Australia<br><br>2-arm, quasi-experimental (1 intervention, 1 control)<br><br>Follow-ups: 1 week, 3 months<br><br>Theory mentioned?<br>No<br><br>Sample: Employees	Waking sitting time (over last 7 days) (O, accelerometer) *<br><br>Worksite sitting time (over last 7 days) (O, accelerometer)*	2/7	Provision of sit-stand workstations and motivational information	N = 18<br>Mean age 33.5y<br>17 female (94%)	N = 13<br>Mean age 39.9y<br>12 female (86%)	Behaviour types targeted: SB, PA<br><br>Primary behaviour change aim: to reduce SB<br><br>Functions: Environmental restructuring<br><br>BCTs: Instruction on how to perform the behaviour; information about social and emotional consequences; restructuring the physical environment; adding objects to the environment	**Very promising**<br><br>*Within-group:* Reduction in workplace sitting and overall waking time sitting at 1 week and 3 months.<br><br>*Between-group:* Greater change in workplace sitting and overall waking time sitting at 1 week and 3 months relative to control group (for which no change was observed).

SB an explicit eligibility criterion?<br>No<br><br>Setting: Worksite<br><br>Total sample at final follow-up: N = 31							
Barwais et al (2013)<br><br>Australia<br><br>2-arm RCT (1 intervention, 1 control)<br><br>Follow-up: 4 weeks only<br><br>Theory mentioned?<br>No<br><br>Sample: Office workers and	Waking sitting time (over last 7 days) (SR) *	4/7	Provision of an activity monitor for PA feedback	N = 18<br>Mean age 28.7y males, 29.5y females<br>6 females (33%)	N = 15<br>Mean age 26.1y males, 27.2y females<br>5 females (67%)	Behaviour types targeted: SB, PA<br><br>Primary behaviour change aim: to reduce SB<br><br>Functions: Enablement, education<br><br>BCTs: Goal-setting (outcome); Feedback on behaviour; Social support (unspecified); Adding objects to environment	**Very promising**<br><br>*Within-group:* Reduction in sitting time.<br><br>*Between-group:* Greater change in sitting time relative to control group (for which no change was observed).

students<br><br>SB an explicit eligibility criterion?<br>Yes<br><br>Setting:<br>Community<br><br>Total sample at follow-up:<br>N = 33							
Burke et al (2013) (also Burke et al 2010) †<br><br>Australia<br><br>2-arm RCT (1 intervention, 1 control)<br><br>Follow-up: 6 months only<br><br>Theory mentioned? Yes (Social Cognitive	Waking sitting time (over a typical week) (SR) *	4/7	Multifaceted intervention promoting PA and healthy diet	N = 176<br>Mean age 65.8y<br>83 females (47%)	N = 199<br>Mean age 65.8y<br>98 females (49%)	Behaviour types targeted: PA, diet<br><br>Primary behaviour change aim: to increase PA (and improve diet)<br><br>Function:<br>Enablement<br><br>BCTs: Goal setting (outcome); Feedback on behaviour; Self-monitoring of behaviour; Social support (unspecified); Adding objects to the environment	**Very promising**<br><br>*Within-group:*<br>Reduction in sitting time.<br><br>*Between-group:*<br>Greater change in sitting time relative to control group (for which no change was observed).

Theory)<br><br>Sample: Older adults<br><br>SB an explicit eligibility criterion?<br>No<br><br>Setting: Home-based<br><br>Total sample at follow-up:<br>N = 375							
Chang et al (2013)<br><br>USA<br><br>2-arm quasi-experiment (2 interventions)<br><br>Follow-up: 8 weeks only<br><br>Theory mentioned?<br>Yes	Waking sitting time (over last 7 days) (SR) *	2/7	<u>Intervention 1:</u><br>8-week weekly empowerment course in healthy lifestyle<br><br><u>Intervention 2:</u><br>Lectures on causes, signs and symptoms of hypertension	<u>Intervention 1 (empowerment) :</u><br>N = 27<br>Mean age 66.7y<br>16 female (59%)<br><br><u>Intervention 2 (education):</u><br>N = 21<br>Mean age 66.0y<br>11 female (52%)	N/A	<u>Intervention 1 (empowerment):</u><br>Behaviour types targeted: SB, PA<br><br>Joint primary behaviour change aims: to reduce SB and increase PA<br><br>Functions: Education, training enablement<br><br>BCTs: Goal setting (behaviour); Action planning; Monitoring of behaviour by others without feedback; Self-monitoring of	<u>Intervention 1:</u><br>**Very promising**<br><br>*Within-group:*<br>Reduction in sitting time.<br><br>*Between-group:*<br>Greater reduction in sitting than Intervention 2.<br><br><u>Intervention 2:</u><br>**Non-promising**<br><br>*Within-group:*

(Empowerment Theory)<br><br>Sample: Older adults with hypertension<br><br>SB an explicit eligibility criterion?<br>No<br><br>Setting: Community<br><br>Total sample at follow-up:<br>N = 48						behaviour; Social support (unspecified); Instruction on how to perform the behaviour; Information about health consequences; Behavioural practice/rehearsal; Graded tasks; Credible source; Adding objects to the environment<br><br><u>Intervention 2 (education):</u><br>Behaviour types targeted: Unclear<br><br>Primary behaviour change aim: Unclear<br><br>Functions: Education<br><br>BCTs: Social support (unspecified); Credible source	Unclear.<br><br>*Between-group:*<br>Lesser reduction in sitting relative to Intervention 1.
De Cocker et al (2012) ‡<br><br>Netherlands<br><br>2-arm RCT (2 interventions)<br><br>Follow-up: 3 months only	Waking sitting time (over a typical week) (SR) *	5/7	<u>Intervention 1:</u> Pedometer and tailored feedback<br><br><u>Intervention 2:</u> Pedometer only	<u>Intervention 1 (pedometer + feedback):</u><br>N = 32<br>Mean age 46.6y<br>15 females (47%)<br><br><u>Intervention 2 (pedometer only):</u><br>N = 37	N/A	<u>Intervention 1 (pedometer + feedback):</u><br>Behaviour types targeted: PA<br><br>Primary behaviour change aim: to increase PA<br><br>Functions: Education, enablement, persuasion<br><br>BCTs: Goal setting (behaviour); Problem solving; Action	<u>Intervention 1:</u> **Non-promising**<br><br>*Within-group:*<br>No change in sitting time.<br><br>*Between-group:*<br>No difference in sitting time change relative to Intervention 2.

Theory mentioned?<br>Yes (Transtheoretical Model, Theory of Planned Behaviour)<br><br>Sample: Patients/general public<br><br>SB an explicit eligibility criterion?<br>No<br><br>Setting: Community<br><br>Total sample at follow-up:<br>N = 69				Mean age 47.7y<br>16 females (43%)		planning; Feedback on behaviour; Social support (practical); Instruction on how to perform the behaviour; Information about health consequences; Graded tasks; Adding objects to environment; Verbal persuasion about capability<br><br><u>Intervention 2 (pedometer only):</u><br>Behaviour types targeted: PA<br><br>Primary behaviour change aim: to increase PA<br><br>Function: Enablement<br><br>BCTs: Goal setting (behaviour); Feedback on behaviour; Adding objects to environment	<u>Intervention 2:</u><br>**Non-promising**<br><br>*Within-group:*<br>No change in sitting time.<br><br>*Between-group:*<br>No difference in sitting time change relative to Intervention 1.
Dewa et al (2009)<br><br>Canada<br><br>Quasi-experimental	Waking sitting time (over last 7 days) (SR) *	0/7	Provision of pedometer	N = 22<br>Mean age 43.4y<br>Gender NR (all participants = 25 females, 3 males)	N = 6<br>Mean age 53.8y<br>Gender NR (all participants = 25 females, 3 males)	Behaviour types targeted: PA, SB<br><br>Primary behaviour change aim: to increase PA and reduce SB<br><br>Function:	**Very promising**<br><br>*Within-group:*<br>Reduction in sitting time.<br><br>*Between-group:*

(1 intervention, 1 control)<br><br>Follow-up: 4 weeks only<br><br>Theory mentioned? No<br><br>Sample: Employees<br><br>SB an explicit eligibility criterion? No<br><br>Setting: Worksite<br><br>Total sample at follow-up: N = 28						Enablement<br><br>BCTs: Self-monitoring of behaviour; Adding objects to environment	Greater change in sitting time relative to control group (for which no change was observed).
Dunn et al (1998) (also: Dunn et al, 1997; Kohl et al, 1998)<br><br>USA	Waking sitting time (over last 7 days) (SR) *	2/7	<u>Intervention 1:</u> Structured exercise classes<br><br><u>Intervention 2:</u> Advice on integrating PA	<u>Intervention 1 (structured):</u> N = 104 Mean age 46.2y 53 females (51%)	N/A	<u>Intervention 1 (structured):</u> Behaviour types targeted: PA<br><br>Primary behaviour change aim: to increase PA<br><br>Functions: Education,	<u>Intervention 1:</u> **Quite promising**<br><br>*Within-group:* Reduction in sitting time.

2-arm RCT (2 interventions)<br><br>Follow-up: 6 months only<br><br>Theory mentioned? Yes (Transtheoretical Model, Social Cognitive Theory)<br><br>Sample: General public<br><br>SB an explicit eligibility criterion? No<br><br>Setting: Unclear<br><br>Total sample at follow-up: N = 214			into lifestyle	<u>Intervention 2 (lifestyle):</u><br>N = 110<br>Mean age 45.9y<br>55 females (50%)		enablement, persuasion, training<br><br>BCTs: Goal setting (behaviour); Action planning; Self-monitoring of behaviour; Social support (unspecified); Graded tasks; Credible source; Social reward<br><br><u>Intervention 2 (lifestyle):</u><br>Behaviour types targeted: PA<br><br>Primary behaviour change aim: to increase PA<br><br>Functions: Persuasion, enablement, education<br><br>BCTs: Goal setting (behaviour); Problem solving; Action planning; Commitment; Social support (unspecified); Information about health consequences; Prompts/cues; Behaviour substitution; Self-reward	*Between-group*: No difference relative to Intervention 2.<br><br><u>Intervention 2:</u> **Quite promising**<br><br>*Within-group:* Reduction in sitting time.<br><br>*Between-group*: No difference relative to Intervention 1.

Ellegast et al (2012) ‡<br><br>Germany?<br><br>2-arm RCT (1 intervention, 1 control)<br><br>Follow-up: weekly for 12 weeks<br><br>Theory mentioned? No<br><br>Sample: Office workers<br><br>SB an explicit eligibility criterion? No<br><br>Setting: Worksite<br><br>Total sample at final follow-up: N =	Worksite sitting time (over last 7 days) (SR) *	1/7	Provision of sit-stand workstations and other intervention measures (unclear)	N = 13<br>Age NR<br>Total sample: 6 females (24%), 19 males (76%)	N = 12<br>Age NR<br>Total sample: 6 females (24%), 19 males (76%)	Behaviour types targeted: PA<br><br>Primary behaviour change aim: to increase PA<br><br>Functions: Persuasion, incentivisation, environmental restructuring, modelling, enablement<br><br>BCTs: Goal setting (behaviour); Discrepancy between current behaviour and goal; Feedback on behaviour; Self-monitoring of behaviour; Social support (unspecified); Social support (practical); Instruction on how to perform the behaviour; Social comparison; Behavioural practice/rehearsal; Behaviour substitution; Material incentive (behaviour); Material reward (behaviour); Restructuring the physical environment; Restructuring the social environment; Adding objects to the environment	**Very promising**<br><br>*Within-group:* Reduction in sitting time (at 12 weeks – changes at other weeks unclear).<br><br>*Between-group:* Greater reduction in sitting time (at 12 weeks) relative to control group.

25							
Evans et al (2012) † ‡<br><br>UK<br><br>2-arm RCT (2 interventions)<br><br>Follow-up: 5 days only<br><br>Theory mentioned? No<br><br>Sample: Office workers<br><br>SB an explicit eligibility criterion? Yes<br><br>Setting: Worksite<br><br>Total sample at follow-up: N = 28	Worksite sitting time (last 5 days) (O, accelerom eter)<br><br>Worksite sitting events per day (last 5 days) (O, accelerom eter) *<br><br>Worksite sitting events (last 5 days) (O, accelerom eter)<br><br>Duration of prolonged worksite sitting events (last 5 days) (O,	4/7	Intervention 1 (Education + Point of Choice):<br>Educational talk on health risks of sitting and computer reminders to stand every 30mins<br><br>Intervention 2 (Education):<br>Educational talk on health risks of sitting	Intervention 1 (education + PoC):<br>N = 14<br>Mean age 49y<br>11 females (79%)<br><br>Intervention 2 (education):<br>N = 14<br>Mean age 39y<br>11 females (79%)	N/A	Intervention 1 (education + PoC)<br>Behaviour types targeted: SB<br><br>Primary behaviour change aim: To reduce SB<br><br>Functions:<br>Education, Enablement, Environmental restructuring<br><br>BCTs: Goal setting (behaviour); Action planning; Instruction on how to perform behaviour; Information about health consequences; Prompts/cues; Behaviour substitution<br><br>Intervention 2 (education)<br>Behaviour types targeted: SB<br><br>Primary behaviour change aim: To reduce SB<br><br>Functions: Education<br><br>BCTs: Goal setting (behaviour); Action planning; Instruction on how to perform behaviour; Information about health consequences; Behaviour	Intervention 1:<br>**Very promising**<br><br>*Within-group:*<br>Reduction in number and duration of bouts of prolonged sitting.<br><br>*Between-group:*<br>Greater reduction in number and duration of bouts of prolonged sitting relative to Intervention 2.<br><br>Intervention 2:<br>**Non-promising**<br><br>*Within-group:*<br>*Increase* in number and duration of bouts of prolonged sitting. No change on other SB indices.<br><br>*Between-group:*

	accelerometer) *					substitution	Greater reduction observed in reduction in Intervention 1 than Intervention 2.
Fitzsimons et al (2012) (also Baker et al, 2008; Fitzsimons et al, 2008)<br><br>UK<br><br>2-arm RCT (2 interventions)<br><br>Follow-up: 3 months (intervention 1 only), 12 months (both interventions)<br><br>Theory mentioned? Yes (Transtheoret	Waking weekday sitting time (over last 7 days) (SR) *<br><br>Waking weekend sitting time (over last 7 days) (SR) *<br><br>Total waking sitting time (last 7 days) (SR) *	3/7	<u>Intervention 1:</u><br>Walking programme with provision of pedometer and PA feedback (in first 12 weeks), PA consultation (at 12 weeks), information and feedback at 24- and 36-weeks<br><br><u>Intervention 2:</u><br><u>Walking programme with provision of pedometer and PA feedback (12-24 weeks),</u> feedback at 24- and 36-weeks	<u>Intervention 1:</u><br>N = 39<br>Mean age 47.3y<br>31 females (79%)<br><br><u>Intervention 2:</u><br>N = 40<br>Mean age 51.2y<br>32 females (80%)	N/A	Intervention 1:<br>Behaviour types targeted: PA<br><br>Primary behaviour change aim: To increase PA<br><br>Function: Enablement<br><br>BCTs: Goal setting (behaviour); Problem solving; Action planning; Feedback on behaviour; Self-monitoring of behaviour; Social support (unspecified); Instruction on how to perform the behaviour; Graded tasks; Pros/cons; Adding objects to the environment<br><br><u>Intervention 2:</u><br>Behaviour types targeted: PA<br><br>Primary behaviour change aim: To increase PA	**Intervention 1: Very promising**<br><br>*Within-group:* Reduction in all sitting time indices (at 3 months and 12 months).<br><br>*Between-group:* Greater change in weekday sitting (between baseline and week 12, and between week 24 and 48), and total sitting time (between baseline and week 12) relative to Intervention Group 2.<br><br>**<u>Intervention 2:</u>**

ical Model)<br><br>Sample: General public<br><br>SB an explicit eligibility criterion?<br>No<br><br>Setting: Community<br><br>Total sample at final follow-up:<br>N = 79						Function: Enablement<br><br>BCTs: Goal setting (behaviour); Action planning; Feedback on behaviour; Self-monitoring of behaviour; Social support (unspecified); Instruction on how to perform the behaviour; Graded tasks; Adding objects to the environment	**Non-promising**<br><br>*Within-group:* Unclear.<br><br>*Between-group:* Less change than Group 1.
Fitzsimons et al (2013)<br><br>Scotland, UK<br><br>1-arm pre-post (no control)<br><br>Follow-up: 24 days only<br><br>Theory mentioned?	Waking sedentary time (over last 7 days) (O, accelerometer) *	2/7	Accelerometer feedback and individualised consultation	N = 22<br>Mean age 68y<br>Mostly male (number/% not specified)	N/A	Behaviour types targeted: SB, PA<br><br>Primary behaviour change aim: To reduce SB<br><br>Functions: Enablement, education, training<br><br>BCTs: Goal setting (behaviour); Problem solving; Action planning; Commitment; Feedback on behaviour; Instruction on how to perform	**Quite promising**<br><br>*Within-group:* Reduction in sitting time<br><br>*Between-group:* N/A

No<br><br>Sample: Older adults<br><br>SB an explicit eligibility criterion?<br>No<br><br>Setting: Community<br><br>Total sample at follow-up:<br>N = 22						the behaviour; Information about health consequences; Behavioural practice/rehearsal; Habit formation; Habit reversal; Graded tasks; Pros and cons	
Gilson et al (2009)<br><br>UK, Australia & Spain<br><br>3-arm RCT (2 interventions, 1 control)<br><br>Follow-ups: 1, 5, and 10 weeks<br><br>Theory mentioned?	Worksite sitting time (over 5 days) (SR) *	2/7	Intervention 1:<br>Pedometer and advice to increase route-based walking during work breaks<br><br>Intervention 2:<br>Pedometer and advice to increase incidental walking during work	Intervention 1 (route):<br>N = 60<br>Mean age 42.1y<br>45 female (75%)<br><br>Intervention 2 (incidental):<br>N = 59<br>Mean age 41.0y<br>47 female (80%)	N = 60<br>Mean age 40.8y<br>49 female (82%)	Intervention 1 (route):<br>Behaviour types targeted: PA<br><br>Primary behaviour change aim: To increase PA<br><br>Function: Enablement<br><br>BCTs: Goal setting (behaviour); Self-monitoring of behaviour; Instruction on how to perform behaviour; Graded tasks; Adding objects to the environment<br><br>Intervention 2 (incidental):	Intervention 1:<br>**Non-promising**<br><br>*Within-group:*<br>No change in sitting time (for average change from baseline – change from baseline at each timepoint unclear).<br><br>*Between-group:*<br>No difference in change relative to

No<br><br>Sample: Employees<br><br>SB an explicit eligibility criterion?<br>No<br><br>Setting: Worksite<br><br>Total sample at final follow-up: N = 179						Behaviour types targeted: PA<br><br>Primary behaviour change aim: To increase PA<br><br>Function: Enablement<br><br>BCTs: Goal setting (behaviour); Self-monitoring of behaviour; Instruction on how to perform behaviour; Behaviour substitution; Graded tasks; Adding objects to the environment	Intervention 2 or control group.<br><br><u>Intervention 2:</u><br>**Non-promising**<br><br>*Within-group:*<br>No change in sitting time (for average change from baseline – change from baseline at each timepoint unclear).<br><br>*Between-group:*<br>No difference in change relative to Intervention 1 or control group.

Hansen et al (2012)<br><br>Denmark<br><br>2-arm RCT (1 intervention, 1 control)<br><br>Follow-up: 6 months only<br><br>Theory mentioned? Yes (Transtheoretical Model, Theory of Planned Behaviour)<br><br>Sample: General public<br><br>SB an explicit eligibility criterion? No<br><br>Setting: Online	Waking sitting time *(unclear whether typical or actual)* (SR) *	4/7	Intervention: Website providing tailored PA advice	N = 3555<br>Mean age 50.7y<br>3924 females (65%)	N = 4249<br>Mean age 50.4y<br>4043 females (65%)	Behaviour types targeted: PA<br><br>Primary behaviour change aim: To increase PA<br><br>Function: Enablement<br><br>BCTs: Goal setting (outcome); Problem solving; Feedback on behaviour; Self-monitoring of outcome of behaviour; Biofeedback; Social support (unspecified); Information on social and emotional consequences; Credible source; Restructuring the social environment	**Non-promising**<br><br>*Within-group:*<br>No changes in sitting time.<br><br>*Between-group:*<br>No difference in change relative to control.

Total sample at follow-up:<br>N = 7804							
Healy et al (2013) (also Dunstan et al, 2013; Neuhaus et al, 2014) † ‡<br><br>Australia<br><br>2-arm non-randomised controlled	Worksite sitting time (over 7 days) (O, accelerom eter) *	2/7	Multilevel intervention promoting organizational, environmental and individual-level change to support SB reduction	N = 18<br>Mean age 42.4y<br>17 females (77%)	N = 18<br>Mean age 42.9y<br>7 females (33%)	Behaviour types targeted:<br>SB, PA<br><br>Primary behaviour change aim:<br>To reduce SB<br><br>Function: Enablement, environmental restructuring, training, education<br><br>BCTs: Goal setting (behaviour); Problem solving; Goal setting	**Very promising**<br><br>*Within-group:*<br>Reduction in sitting time.<br><br>*Between-group:*<br>Greater change in intervention group than control group (for which no change was

trial (1 intervention, 1 control)<br><br>Follow-up: 4 weeks only<br><br>Theory mentioned? No<br><br>Sample: Employees<br><br>SB an explicit eligibility criterion? No<br><br>Setting: Worksite<br><br>Total sample at follow-up: N = 36						(outcome); Action planning; Review behaviour goal(s); Discrepancy between current behaviour and goal; Feedback on behaviour; Self-monitoring of behaviour; Social support (unspecified); Social support (practical); Instruction on how to perform the behaviour; Information about health consequences; Prompts/cues; Behavioural practice/rehearsal; Behaviour substitution; Habit formation; Graded tasks; Restructuring the physical environment; Restructuring the social environment; Adding objects to the environment	observed).
John et al (2011)<br><br>USA<br><br>1-arm pre-post (no	Waking sitting time on workday (over 2 workdays) (O	2/7	Provision of treadmill workstations	N = 12<br>Age 46.2y<br>7 females (58%)	N/A	Behaviour types targeted: PA<br><br>Primary behaviour change aim: To increase PA<br><br>Function: Environmental restructuring	**Quite promising**<br><br>*Within-group:* Reduction in sitting time at both 3 & 9 months.

control)<br><br>Follow-ups: 3 & 9 months<br><br>Theory mentioned? No<br><br>Sample: Employees<br><br>SB an explicit eligibility criterion? Yes<br><br>Setting: Worksite<br><br>Total sample at final follow-up: N = 12	accelerometer) *					BCTs: Restructuring the physical environment; Adding objects to the environment	*Between-group:* N/A
Kozey-Keadle et al (2011)<br><br>USA<br><br>1-arm pre-post (no control)	Waking sedentary time (last 7 days) (O; accelerometer) *<br><br>Total	2/7	Provision of information and suggested SB reduction strategies	N = 16<br>Age 46.5y<br>15 female (75%)	N/A	Behaviour types targeted: SB<br><br>Primary behaviour change aim: To reduce SB<br><br>Functions: Education, Enablement	**Quite promising**<br><br>*Within-group:* Reduction in sitting time by both measures.<br><br>*Between-group:*

Follow-up: Between 1-7 days only<br><br>Theory mentioned? No<br><br>Sample: Employees<br><br>SB an explicit eligibility criterion? Yes<br><br>Setting: Worksite<br><br>Total sample at follow-up: N = 16	worksite sedentary time (over 6h period) (O, direct observation) *					BCTs: Goal setting (behaviour); Action planning; Self-monitoring of behaviour; Instruction on how to perform the behaviour; Information about health consequences; Prompts/cues; Behavioural substitution	N/A
Marshall et al (2003a) (also: Marshall et al, 2003b) ***<br><br>Australia<br><br>2-arm RCT (2 interventions	Waking sitting time on weekday (SR) *	7/7	<u>Intervention 1:</u><br>Print-based intervention to increase PA<br><br><u>Intervention 2:</u><br>Web-based intervention to increase PA	<u>Intervention 1 (print):</u><br>N = 87<br>Mean age 43y<br>Gender N NR (47% females in group from which 87 derived)	N/A	<u>Intervention 1 (print):</u><br>Behaviour types targeted: PA<br><br>Primary behaviour change aim: To increase PA<br><br>Function: Enablement<br><br>BCTs: Goal setting (outcome);	<u>Intervention 1:</u><br>**Non-promising**<br><br>*Within-group:*<br>No change in sitting time.<br><br>*Between-group:*<br>No difference in

)<br><br>Follow-up: 10 weeks only<br><br>Theory mentioned? Yes (Transtheoretical Model)<br><br>Sample: Employees<br><br>SB an explicit eligibility criterion? No<br><br>Setting: Worksite<br><br>Total sample at follow-up: N = 156				<u>Intervention 2 (web):</u><br>N = 69<br>Mean age 43y<br>Gender N NR (50% females in group from which 69 derived)		Self monitoring of outcome of behaviour; Social support (unspecified); Prompts/cues<br><br><u>Intervention 2 (web):</u><br>Behaviour types targeted: PA<br><br>Primary behaviour change aim: To increase PA<br><br>Function: Enablement<br><br>BCTs: Goal setting (outcome); Self monitoring of outcome of behaviour; Social support (unspecified); Prompts/cues	change relative to Intervention 2.<br><br><u>Intervention 2:</u><br>**Non-promising**<br><br>*Within-group:*<br>No change in sitting time.<br><br>*Between-group:*<br>No difference in change relative to Intervention 1.
Mazzeo et al (2008)<br><br>USA<br><br>2-arm RCT (1 intervention,	Waking sitting time (over last 7 days) (SR) *	3/7	Series of group sessions on weight loss (diet and PA)	N = 17<br>Total sample:<br>Mean age 40.1y<br>33 (97%) female	N = 17<br>Total sample:<br>Mean age 40.1y<br>33 (97%) female	Behaviour types targeted: PA, diet<br><br>Primary behaviour change aim: weight loss (increased PA, improved diet)	**Very promising**<br><br>*Within-group:*<br>Intervention group reduced their sitting time.

1 control)<br><br>Follow-up:<br>Wave 1 sample: 8 weeks only<br>Wave 2 sample: 12 weeks only (data pooled for analysis)<br><br>Theory mentioned? Yes (Social Cognitive Theory)<br><br>Sample: Parents of overweight children<br><br>SB an explicit eligibility criterion? No<br><br>Setting: Community<br><br>Total sample						Functions:<br>Training, enablement<br><br>BCTs: Social support (unspecified); Identification of self as role model	*Between-group:* Reduction in sitting time in intervention group significantly greater than in control group.

at follow-up:<br>N = 34							
Mutrie et al (2012) (also: Macmillan et al, 2011) †<br><br>Scotland, UK<br><br>2-arm RCT (1 intervention, 1 control) ****<br><br>Follow-up: 12 & 24 weeks ****<br><br>Theory mentioned? Yes (Social Cognitive Theory)<br><br>Sample: Older adults<br><br>SB an explicit eligibility criterion? No	Waking sedentary time (over last 7 days) (O, accelerom eter) *	4/7	Pedometer feedback and individualized consultation	N = 20<br>Mean age 71.6y<br>13 females (65%)	N = 19<br>Mean age 70.0y<br>13 females (68%)	Behaviour types targeted: PA<br><br>Primary behaviour change aim: To increase PA<br><br>Function: Enablement<br><br>BCTs: Goal setting (behaviour); Problem solving; Self-monitoring of behaviour; Social support (unspecified); Instruction on how to perform behaviour; Information on health consequences; Graded tasks; Pros and cons; Adding objects to the environment	**Very promising**<br><br>*Within-group:* Reduction in sedentary time at 12 weeks. ****<br><br>*Between-group:* Greater change in intervention group than control group (no change observed) at 12 weeks. ****

Setting: General practice<br><br>Total sample at final follow-up: N = 39							
Opdenacker & Boen (2008)<br><br>Belgium<br><br>2-arm RCT (2 interventions)<br><br>Follow-up: 3 months only<br><br>Theory mentioned? No<br><br>Sample: Employees<br><br>SB an explicit eligibility criterion? No	Waking sitting time (over last 7 days) (SR) *	3/7	Intervention 1:<br>PA coaching programme with face-to-face support<br><br>Intervention 2:<br>PA coaching programme with telephone support	Intervention 1 (face-to-face):<br>N = 33<br>Mean age 38.8y<br>Gender NR<br><br>Intervention 2 (phone):<br>N = 33<br>Mean age 39.9y<br>Gender NR	N/A	Intervention 1 (face-to-face)<br>Behaviour types targeted: PA<br><br>Primary behaviour change aim: To increase PA<br><br>Function: Enablement<br><br>BCTs:<br>Goal setting (behaviour); Problem solving; Review behaviour goals; Self-monitoring of behaviour; Social support (unspecified); Instructions on how to perform behaviour<br><br>Intervention 2 (phone):<br>Behaviour types targeted: PA<br><br>Primary behaviour change aim: To increase PA<br><br>Function: Enablement	Intervention 1:<br>**Quite promising**<br><br>*Within-group:*<br>Reduction in sitting time.<br><br>*Between-group:*<br>No difference in change relative to Intervention 2.<br><br>Intervention 2:<br>**Quite promising**<br><br>*Within-group:*<br>Reduction in sitting time.<br><br>*Between-group:*<br>No difference in change relative to Intervention 1.

Setting: Worksite<br><br>Total sample at follow-up: N = 66						BCTs: Goal setting (behaviour); Problem solving; Review behaviour goals; Self-monitoring of behaviour; Social support (unspecified); Instructions on how to perform behaviour	
Østeras & Hammer (2006)<br><br>Norway<br><br>1-arm pre-post (no control)<br><br>Follow-up: 6 months only<br><br>Theory mentioned? No<br><br>Sample: Employees<br><br>SB an explicit eligibility criterion? Yes	Waking sitting time *(unclear whether typical or actual)* (SR) *	2/7	Individual consultation about increasing PA	N = 131<br>Mean age 41.2y<br>51 females (39%)	N/A	Behaviour types targeted: PA<br><br>Primary behaviour change aim: To increase PA<br><br>Function: Enablement<br><br>BCTs: Goal setting (behaviour); Action planning; Review behaviour goals; Social support (unspecified)	**Non-promising**<br><br>*Within-group:* No change in sitting time<br><br>*Between-group:* N/A

Setting: Worksite<br><br>Total sample at follow-up: N = 131							
Pronk et al (2012)<br><br>USA<br><br>2-arm non-randomised time-series (1 intervention, 1 control)<br><br>Follow-ups: 5 & 7 weeks<br><br>Theory mentioned? No<br><br>Sample: Employees<br><br>SB an explicit eligibility criterion? Yes	Worksite sitting time (over a typical day) (SR) *	2/7	Provision of sit-stand workstations and supportive organizational policies	N = 23<br>Mean age 38.4y<br>23 females (96%)	N = 10<br>Mean age 44.2y<br>8 females (80%)	Behaviour types targeted: SB<br><br>Primary behaviour change aim: To reduce SB<br><br>Functions: Environmental restructuring, Enablement<br><br>BCTs: Restructuring the physical environment; Restructuring the social environment; Adding objects to the environment	**Very promising**<br><br>*Within-group:* Intervention group decreased workplace sitting at 5 weeks.<br><br>*Between-group:* Greater decrease in workplace sitting at 5-week follow-up among intervention group relative to control (control group *increased* workday sitting at 5 weeks).

Setting: Worksite<br><br>Total sample at follow-up: N = 33							
Shaw & Page (2008)<br><br>USA<br><br>2-arm RCT (2 interventions)<br><br>Follow-up: 90 days only<br><br>Theory mentioned? No<br><br>Sample: Older women already participating in a nutrition and PA programme<br><br>SB an explicit	Waking (?) sitting time (over a typical week) (SR) *	3/7	Intervention 1:<br>Instruction to walk for at least 10mins per day and provision of walking aids if needed (and separate PA and dietary intervention administered to all participants before this treatment)<br><br>Intervention 2:<br>Provision of walking aids if needed (and separate PA and dietary intervention administered to all participants before this	Intervention 1:<br>N = 10<br>Modal age 65-69 (31%)<br>10 female (100%)<br><br>Intervention 2:<br>N = 8<br>Joint modal age <65 & 65-69 (each 18%)<br>8 female (100%)	N/A	Intervention 1:<br>Behaviour types targeted: PA (and diet, as part of intervention administered prior to this one)<br><br>Primary behaviour change aim: To increase PA<br><br>Function: Enablement<br><br>BCTs: Goal setting (behaviour); Action planning; Monitoring of bhvr by others without feedback; Instruction on how to perform behaviour; Graded tasks; Adding objects to the environment<br><br>Intervention 2:<br>Behaviour types targeted: PA (and diet, as part of intervention administered prior to this one)	Intervention 1:<br>**Non-promising**<br><br>*Within-group:*<br>No change in sitting time.<br><br>*Between-group:*<br>No change relative to Intervention 2.<br><br>Intervention 2:<br>**Non-promising**<br><br>*Within-group:*<br>No change in sitting time.<br><br>*Between-group:*<br>No change relative to Intervention 1.

eligibility criterion?<br>No<br><br>Setting: Community<br><br>Total sample at follow-up:<br>N = 18			treatment)			Primary behaviour change aim: To increase PA<br><br>Function: Enablement<br><br>BCTs: Adding objects to the environment, instruction on how to perform behaviour	
Spittaels et al (2007) (also Spittaels & De Bourdeaudhuij, 2007) ‡<br><br>Belgium<br><br>3-arm cluster RCT (2 intervention, 1 control)<br><br>Follow-up: 6 months only<br><br>Theory mentioned? Yes (Transtheoretical Model, Theory of	Waking sitting time *(unclear whether typical or actual)* (SR) *	3/7	<u>Intervention 1 (one-off feedback):</u><br>Website providing one-off PA feedback<br><br><u>Intervention 2 (repeated feedback):</u><br>Website providing repeated PA feedback	<u>Intervention 1 (one-off feedback):</u><br>N = 103<br>Mean age 43.3y<br>67 females (65%)<br><br><u>Intervention 2 (repeated feedback):</u><br>N = 78<br>Mean age 39.6y<br>52 females (67%)	N = 104<br>Mean age 40.7y<br>70 females (67%)	<u>Intervention 1 (one-off feedback):</u><br>Behaviour types targeted: PA<br><br>Primary behaviour change aim: To increase PA<br><br>Function:<br>Enablement<br><br>BCTs: Goal setting (outcome); Feedback on behaviour; Social support (unspecified); Social reward<br><br><u>Intervention 2 (repeated feedback):</u><br>Behaviour types targeted: PA<br><br>Primary behaviour change aim: To increase PA	<u>Intervention 1:</u><br>**Very promising**<br><br>*Within-group:* Reduction in sitting on both weekdays and weekends.<br><br>*Between-group:* Greater change than control group. No difference relative to Intervention 2.<br><br><u>Intervention 2:</u><br>**Very promising**<br><br>*Within-group:* Reduction in sitting on both

Planned Behaviour)<br><br>Sample: School staff and parents<br><br>SB an explicit eligibility criterion? No<br><br>Setting: Online<br><br>Total sample at follow-up: N = 285						Function: Enablement<br><br>BCTs: Goal setting (outcome); Feedback on behaviour; Social support (unspecified); Social reward	weekdays and weekends.<br><br>*Between-group:* Greater change than control group. No difference relative to Intervention 1.
Verweij et al (2012) †<br><br>Netherlands<br><br>2-arm cluster RCT (2 interventions)<br><br>Follow-up: 6 months only<br><br>Theory	Waking sitting time (over a typical week) (SR) *	4/7	<u>Intervention 1:</u> Advising employers on environmental risk factors for employee weight gain, and employees on individual-level risk factors and providing advice on PA, SB, and diet	<u>Intervention 1</u><br>N = 230<br>Mean age 46y<br>87 females (38%)<br><br><u>Intervention 2</u><br>N = 224<br>Mean age 48y<br>78 females (35%)	N/A	<u>Intervention 1:</u><br>Behaviour types targeted: PA, SB, diet<br><br>Primary behaviour change aim: Weight loss (not behaviour)<br><br>Function: Enablement<br><br>BCTs: Goal setting (behaviour); Problem solving; Goal setting (outcome); Action planning; Review outcome goals; Self-monitoring of behaviour; Self-	<u>Intervention 1:</u><br>**Very promising**<br><br>*Within-group:* Reduction in weekday sitting time at work.<br><br>*Between-group:* Greater SB change relative to Intervention 2.<br><br><u>Intervention 2:</u>

mentioned?<br>No<br><br>Sample:<br>Employees<br><br>SB an explicit eligibility criterion?<br>No<br><br>Setting:<br>Worksite<br><br>Total sample at follow-up:<br>N = 454			<u>Intervention 2:</u><br>Individual health risk appraisal and advice			monitoring of outcomes of behaviour; Social support (unspecified); Pros and cons; Adding objects to the environment<br><br><u>Intervention 2:</u><br>Behavioural types targeted: Unclear<br><br>Primary behaviour change aim: Weight loss (not behaviour)<br><br>Function: Unclear<br><br>BCTs: None coded	**Non-promising**<br><br>*Within-group:*<br>No change in SB.<br><br>*Between-group:*<br>Less SB change relative to Intervention 1.

Abbreviations: BCT = Behaviour Change Technique. N/A = Not applicable. NR = Not reported. O = Objective. PA = Physical activity. RCT = Randomised controlled trial. SB = Sedentary behaviour. SR = Self-report.

Ns denote sample size for those entered into analysis of changes in sedentary behaviour, at the last follow-up point (or last point at which changes from baseline were reported), so may differ from Ns reported in sample descriptions within published papers. Demographics refer to those recruited at baseline, so total N may not match Ns reported in this table.

† Information also extracted from record in public trial database.
‡ Information also extracted from additional material sent by author.
* Measures marked with two asterisks indicate those used as indicators of intervention promise (final column of this table).
** 'Follow-up' refers to point(s) at which SB was measured, so excludes time points at which SB was not measured.

*** Data reported from Marshall et al (2003a) relate to participants who received *and read* the information given to them, as this represents closest fidelity to the intervention treatments.
**** Mutrie et al (2012) used a waiting-list control group design, administering the intervention to the control group at 12 weeks, and comparing groups at 24 weeks. Given both groups had received the intervention by 24-weeks, intervention promise cannot be reliably estimated and so is not reported here.
