# Supplementary material for: How to reduce sitting time? A review of behaviour change strategies used in sedentary behaviour reduction interventions among adults
Source: Health Psychol Rev. 2015 Sep 16;10(1):89–112. doi: 10.1080/17437199.2015.1082146 (PMC4743603; doi:10.1080/17437199.2015.1082146)
Supplement: Supplemental Table_3.pdf [file rhpr_a_1082146_sm1772.pdf]

Supplemental Table 3. Study quality.

Source	*Intervention setting*	*Quality criteria*							*Quality score*
		Method of randomization described?	Treatment allocation concealed?	Groups similar at baseline?	Eligibility criteria specified?	Outcome assessor blinded?	Point estimate and evidence of validity for focal SB measure?	Intention-to-treat analysis?	
Aittasalo 2004	Worksite	Yes	Unclear	No	Yes	No	Yes	No	3
Alkhajah 2012	Worksite	No	No	No	Yes	Unclear	Yes	No	2
Barwais 2013	Non-worksite	Yes	Unclear	Yes	Yes	Unclear	Yes	No	4
Burke 2013	Non-worksite	Yes	Unclear	Yes	Yes	Unclear	Yes	No	4
Chang 2013	Non-worksite	No	No	Yes	Yes	Unclear	No	No	2
De Cocker 2012	Non-worksite	Yes	Unclear	Yes	Yes	Unclear	Yes	Yes	5
Dewa 2009	Worksite	N/A	N/A	N/A	No	N/A	Unclear	No	0
Dunn 1998	Non-worksite	Yes	Unclear	No	Yes	Unclear	Unclear	No	2
Ellegast 2012	Worksite	Yes	Unclear	Unclear	No	Unclear	No	No	1
Evans 2012	Worksite	Yes	No	Yes	Unclear	Yes	Yes	No	4
Fitzsimons 2012	Non-worksite	Yes	No	Unclear	Yes	Yes	No	No	3
Fitzsimons 2013	Non-worksite	N/A	N/A	N/A	Yes	N/A	Yes	No	2
Gilson 2009	Worksite	Yes	Unclear	Yes	No	No	No	No	2
Hansen 2012	Non-worksite	Yes	No	No	Yes	No	Yes	Yes	4
Healy 2013	Worksite	N/A	Unclear	Yes	Yes	No	No	No	2
John 2011	Worksite	N/A	N/A	N/A	Yes	N/A	Yes	No	2
Kozey-Keadle 2011	Worksite	N/A	N/A	N/A	Yes	N/A	Yes	No	2
Marshall 2003	Worksite	Yes	Yes	Yes	Yes	Yes	Yes	Yes	7
Mazzeo 2008	Non-worksite	Yes	Unclear	No	Yes	Unclear	Yes	No	3

Mutrie 2012	Non-worksite	Yes	Unclear	Unclear	Yes	Unclear	Yes	Yes	4
Opdenacker 2008	Worksite	Yes	Unclear	Yes	Yes	No	No	No	3
Østeras 2006	Worksite	N/A	N/A	N/A	Yes	N/A	Yes	No	2
Pronk 2012	Worksite	No	Unclear	Yes	Yes	Unclear	No	No	2
Shaw 2008	Non-worksite	Yes	Unclear	Unclear	Yes	Unclear	Yes	No	3
Spittaels 2007	Non-worksite	Yes	Unclear	Unclear	Yes	Unclear	Yes	Yes	3
Verweij 2012	Worksite	Yes	Unclear	Yes	Yes	Unclear	Yes	No	4
