# Supplementary material for: How to reduce sitting time? A review of behaviour change strategies used in sedentary behaviour reduction interventions among adults
Source: Health Psychol Rev. 2015 Sep 16;10(1):89–112. doi: 10.1080/17437199.2015.1082146 (PMC4743603; doi:10.1080/17437199.2015.1082146)
Supplement: Supplemental Table_4.pdf [file rhpr_a_1082146_sm1770.pdf]

Supplemental Table 4. Characteristics of worksite-based interventions, by intervention promise.

Characteristics	*Interventions*				*Promise ratio for intervention functions / behaviour change techniques **
	Very promising (n = 7)	*Quite promising (n = 5)*	*Non-promising (n = 8)*	*All (n = 20)*	
Primary behaviour change aim					
To reduce sedentary behaviour	4	1	1	*6*	
Joint: To reduce sedentary behaviour and increase physical activity	1	0	0	*1*	
To increase physical activity	1	4	6	*11*	
Other	1	0	1	*2*	
Intervention functions					
**Education**	**2**	**2**	**1**	***5***	***4.0***
Persuasion	1	0	0	*1*	-
Incentivisation	1	0	0	*1*	-
Training	1	0	0	*1*	-
**Environmental restructuring**	**5**	**1**	**0**	***6***	**-**
Modelling	1	0	0	*1*	-
Enablement	6	4	6	*16*	*1.7*
Behaviour change techniques					
Goal setting (behaviour)	4	4	5	*13*	*1.6*
**Problem solving**	**2**	**2**	**0**	***4***	**-**
Goal setting (outcome)	2	0	2	*4*	*1.0*
Action planning	3	2	3	*8*	*1.7*
**Review behavioural goals**	**1**	**3**	**2**	***6***	***2.0***
**Discrepancy between current behaviour and goal**	**2**	**0**	**0**	***2***	**-**
Review outcome goals	1	0	0	*1*	-
**Feedback on behaviour**	**2**	**0**	**0**	***2***	**-**

**Self-monitoring (behaviour)**	**4**	**3**	**2**	***9***	***3.5***
Self-monitoring (outcome)	1	0	2	*3*	*0.5*
Feedback on outcomes	0	1	0	*1*	-
Social support (unspecified)	3	3	4	*10*	*1.5*
**Social support (practical)**	**2**	**0**	**0**	***2***	**-**
**Instruction on how to perform behaviour**	**4**	**3**	**3**	***10***	***2.3***
**Information on health consequences**	**2**	**2**	**2**	***6***	***2.0***
Information on social and emotional consequences	1	0	0	*1*	-
**Social comparison**	**1**	**1**	**0**	***2***	**-**
Prompts / cues	2	1	2	*5*	*1.5*
**Behavioural practice / rehearsal**	**2**	**0**	**0**	***2***	**-**
**Behaviour substitution**	**3**	**1**	**2**	***6***	***2.0***
Habit formation	1	0	0	*1*	-
Graded tasks	1	0	2	*3*	*0.5*
Pros and cons	1	0	0	*1*	-
Material incentive for behaviour	1	0	0	*1*	-
Material reward for behaviour	1	0	0	*1*	-
**Restructuring the physical environment**	**4**	**1**	**0**	***5***	**-**
**Restructuring the social environment**	**3**	**0**	**0**	***3***	**-**
**Adding objects to the environment**	**6**	**1**	**2**	***9***	***3.5***

* Promise ratio denotes the number of very or quite-promising interventions in which an intervention function or behaviour change technique featured, divided by the number of non-promising interventions in which it featured. Promise ratios only calculable for functions or techniques used in both promising and non-promising interventions. Rows in bold denote functions or techniques associated with a promise ratio of 2 or above, or used exclusively in promising interventions, and which featured in at least two interventions.
