# Supplementary material for: How to reduce sitting time? A review of behaviour change strategies used in sedentary behaviour reduction interventions among adults
Source: Health Psychol Rev. 2015 Sep 16;10(1):89–112. doi: 10.1080/17437199.2015.1082146 (PMC4743603; doi:10.1080/17437199.2015.1082146)
Supplement: Supplemental Table_5.pdf [file rhpr_a_1082146_sm1766.pdf]

Supplemental Table 5. Description of studies excluded because sedentary behaviour was inferred from time spent in typically-sedentary activities

Reference and misc details	*Sedentary behaviour measures*	*Quality*	*Description of intervention treatment(s)*	*Intervention group(s) description*	*Control group description*	*Intervention content*	*Promise*
Fitzgibbon et al (2005)<br><br>USA<br><br>2-arm RCT (2 interventions) with 2 cohorts<br><br>Follow-up: 20 weeks only<br><br>Theory mentioned? Yes (Social Cognitive Theory)<br><br>Sample: Overweight or obese black women<br><br>SB an explicit eligibility	TV viewing (hours) in a typical day (SR)	3/7	Intervention 1, cohort 1:<br>Series of group discussions<br><br>Intervention 1, cohort 2:<br>Series of group discussions<br><br>Intervention 2, both cohorts:<br>Newsletters about various health topics (including smoking, but not SB, PA, or diet)	Intervention 1, cohort 1:<br>N = 12<br>Mean age (cohort 1 combined) 44.4y<br>12 female (100%)<br><br>Intervention 1, cohort 2:<br>N = 14<br>Mean age (cohort 2 combined) 45.1y<br>14 female (100%)<br><br>Intervention 2, cohort 1:<br>N = 13<br>Mean age (cohort 1 combined) 44.4y<br>13 female (100%)	N/A	Intervention 1 (both cohorts combined):<br>Behaviour types targeted: PA, fat consumption, breast self-examination<br><br>Primary behaviour change aim: To decrease weight and fat intake, and increase PA and BSE<br><br>Function: Enablement<br><br>BCTs: Goal setting (behaviour); Problem solving; Goal setting (outcome); Review outcome goals; Self-monitoring of behaviour; Self-monitoring of outcome of behaviour; Social support (unspecified); Instruction on how to perform the behaviour; Information about antecedents; Information about health consequences; Demonstration of the behaviour; Behavioural	Intervention 1:<br>**Non-promising**<br><br>*Within-group:*<br>No change in TV viewing.<br><br>*Between-group:*<br>No difference in change relative to Intervention 2.<br><br>Intervention 2:<br>**Non-promising**<br><br>*Within-group:*<br>No change in TV viewing.<br><br>*Between-group:*<br>No difference in change relative to Intervention 1.

criterion?<br>No<br><br>Setting: Community (cohort 1) / university (cohort 2)<br><br>Total sample at follow-up:<br>N = 57				<u>Intervention 2, cohort 2:</u><br>N = 18<br>Mean age (cohort 2 combined) 45.1y<br>18 female (100%)		practice/rehearsal; Pros/cons; Avoidance/reducing exposure to cues for the behaviour<br><br><u>Intervention 2 (both cohorts combined)</u><br>Behaviour types targeted: Smoking<br><br>Primary behaviour change aim: Unclear<br><br>Function: Education<br><br>BCTs: None coded	
French et al (2011)<br><br>USA<br><br>2-arm cluster RCT (1 intervention, 1 control)<br><br>Follow-up: 12 months only<br><br>Theory mentioned?<br>No	TV-viewing (incl computer use), hours per day *(unclear whether actual or typical)* (SR)	2/7	Multicomponent intervention to promote weight-loss behaviours in the family within the home	N = 43<br>Mean age NR (total sample 41y)<br>Gender NR	N = 44<br>Mean age NR (total sample 41y)<br>Gender NR	Behaviour types targeted: SB, PA, dietary intake<br><br>Primary behaviour change aim: No explicit primary behaviour change aim<br><br>Functions: Enablement, restriction, incentivisation, training, environmental restructuring<br><br>BCTs: Goal setting (behaviour); Problem solving; Goal setting (outcome); Commitment; Self-monitoring of behaviour; Social support (unspecified);	**Quite promising**<br><br>*Within-group:* Apparent reduction in TV-viewing, but unclear whether significant.<br><br>*Between-group:* Greater reduction among intervention group relative to control group.

Sample: General public<br><br>SB an explicit eligibility criterion? Yes (TV viewing >10h/w)<br><br>Setting: Home-based<br><br>Total sample at follow-up: N = 87						Instruction on how to perform the behaviour; Behavioural practice/rehearsal; Behavioural substitution; Restructuring the physical environment; Avoidance/reducing exposure to cues for the behaviour; Adding objects to the environment	
Jago et al (2013)<br><br>UK<br><br>2-arm RCT (1 intervention, 1 control)<br><br>Follow-ups: 8 & 16 weeks<br><br>Theory mentioned? Yes (Self-	Screen-viewing, typical hrs per weekend day (SR)<br><br>Screen-viewing typical hrs per weekday (SR)	2/7	Parenting programme promoting reduction of screen time for parents and children	N = 22<br>Age NR<br>22 females (100%)	N = 11<br>Age NR<br>22 females (96%)	Behaviour types targeted: SB, PA<br><br>Primary behaviour change aim: To reduce SB and increase PA<br><br>Function: Enablement, training, modeling<br><br>BCTs: Goal setting (behaviour); Problem solving; Commitment; Social support (unspecified); Behavioural experiments; Information on social and emotional consequences; Pros	**Unclear**<br><br>*Within- and between-group:* Weekday viewing appeared to decrease for intervention group, but increase for control group.<br><br>Weekend viewing appeared to decrease more in

Determination Theory)<br><br>Sample: Parents<br><br>SB an explicit eligibility criterion?<br>No<br><br>Setting: Community & home-based<br><br>Total sample at follow-up*: N = 33						and cons; Restructuring the social environment	intervention group than in control group.<br><br>Statistical significance of changes not reported.
Otten et al (2009)<br><br>USA<br><br>2-arm RCT (1 intervention, 1 control)<br><br>Follow-up: 3 weeks<br><br>Theory mentioned?	TV viewing, hrs per day (O)	3/7	Device limiting TV viewing	N = 20<br>Mean age 42.8y<br>14 females (70%)	N = 16<br>Mean age 42.4y<br>11 females (69%)	Behaviour types targeted: SB<br><br>Primary behaviour change aim: To reduce SB<br><br>Function: Restriction<br><br>BCTs: Goal setting (behaviour); Restructuring the physical environment	**Very promising**<br><br>*Within-group:* Intervention group decreased TV viewing.<br><br>*Between-group:* Intervention group achieved greater reduction in TV viewing than did control group.

No<br><br>Sample: Overweight and obese adults<br><br>SB an explicit eligibility criterion? Yes (TV viewing 3-8h/d)<br><br>Setting: Home<br><br>Total sample at follow-up: N = 36							
Steeves et al (2012)<br><br>USA<br><br>2-arm RCT (2 interventions, no control)<br><br>Follow-up: 3 & 6 months<br><br>Theory	TV viewing, actual hrs per day (SR)	4/7	<u>Intervention 1 (commercial stepping):</u><br>Instruction to briskly step on the spot or walk continuously during commercial breaks in 90+ mins' TV viewing	<u>Intervention 1 (commercial stepping):</u><br>N = 29<br>Mean age 53.8y<br>24 females (83%)<br><br><u>Intervention 2 (walking):</u><br>N = 29<br>Mean age 50.2y<br>22 females	N/A	<u>Intervention 1 (commercial stepping):</u><br>Behaviour types targeted: PA, SB<br><br>Primary behaviour change aim: To increase PA and reduce SB<br><br>Functions: Environmental restructuring, enablement<br><br>BCTs: Goal setting (behaviour); Problem solving; Action	<u>Intervention 1:</u><br>**Quite promising**<br><br>*Within-group:*<br>Reduction in TV viewing time at 6 months.<br><br>*Between-group:*<br>No differences (at 3 or 6 months).<br><br><u>Intervention 2:</u>

mentioned?<br>No<br><br>Sample: Overweight and sedentary members of general public<br><br>SB an explicit eligibility criterion?<br>Yes (TV viewing ≥14h/w)<br><br>Setting: Home<br><br>Total sample at follow-up*:<br>N = 58			<u>Intervention 2 (walking):</u><br>Instruction to walk for at least 30min at least 5 days/week	(76%)		planning; Self-monitoring of behaviour; Social support (unspecified); Instruction on how to perform behaviour; Prompts/cues; Behavioural practice/rehearsal; Graded tasks<br><br><u>Intervention 2 (walking):</u><br>Behaviour types targeted:<br>PA<br><br>Primary aim: To increase PA<br><br>Function: Enablement<br><br>BCTs: Goal setting (behaviour); Problem solving; Action planning; Self-monitoring of behaviour; Social support (unspecified); Instruction on how to perform behaviour; Graded tasks	**Quite promising**<br><br>*Within-group:*<br>Reduction in TV viewing time at 6 months.<br><br>*Between-group:*<br>No differences (at 3 or 6 months).

Abbreviations: BCT = Behaviour Change Technique. N/A = Not applicable. NR = Not reported. O = Objective. PA = Physical activity. RCT = Randomised controlled trial. SB = Sedentary behaviour. SR = Self-report.

Ns denote sample size for those entered into analysis of changes in sedentary behaviour, at the last follow-up point (or last point at which changes from baseline were reported), so may differ from Ns reported in sample descriptions within published papers. Demographics refer to those recruited at baseline, so total N may not match Ns reported in this table.

* ‘Follow-up’ refers to the final point at which SB was measured. This differs from the final study measurement point where SB was not measured at the final study measurement point.
